# Pyridazinones and Structurally Related Derivatives with Anti-Inflammatory Activity

**DOI:** 10.3390/molecules27123749

**Published:** 2022-06-10

**Authors:** Niccolo Cantini, Igor A. Schepetkin, Nadezhda V. Danilenko, Andrei I. Khlebnikov, Letizia Crocetti, Maria Paola Giovannoni, Liliya N. Kirpotina, Mark T. Quinn

**Affiliations:** 1 Department of Microbiology and Cell Biology, Montana State University, Bozeman, MT 59717, USA; niccolo.cantini@uantwerpen.be (N.C.); igor@montana.edu (I.A.S.); liliya@montana.edu (L.N.K.); 2 Department of Medicinal Chemistry, University of Antwerp, 2610 Antwerp, Belgium; 3 NEUROFARBA, Pharmaceutical and Nutraceutical Section, University of Florence, 50019 Sesto Fiorentino, Italy; letizia.crocetti@unifi.it (L.C.); mariapaola.giovannoni@unifi.it (M.P.G.); 4 Kizhner Research Center, Tomsk Polytechnic University, Tomsk 634050, Russia; nadezhda.dani@gmail.com (N.V.D.); aikhl@chem.org.ru (A.I.K.)

**Keywords:** anti-inflammatory, pyridazinone, N-formyl peptide receptor, nuclear factor-κB, monocyte/macrophage, binary classification tree

## Abstract

Persistent inflammation contributes to a number of diseases; therefore, control of the inflammatory response is an important therapeutic goal. In an effort to identify novel anti-inflammatory compounds, we screened a library of pyridazinones and structurally related derivatives that were used previously to identify N-formyl peptide receptor (FPR) agonists. Screening of the compounds for their ability to inhibit lipopolysaccharide (LPS)-induced nuclear factor κB (NF-κB) transcriptional activity in human THP1-Blue monocytic cells identified 48 compounds with anti-inflammatory activity. Interestingly, 34 compounds were FPR agonists, whereas 14 inhibitors of LPS-induced NF-κB activity were not FPR agonists, indicating that they inhibited different signaling pathways. Further analysis of the most potent inhibitors showed that they also inhibited LPS-induced production of interleukin 6 (IL-6) by human MonoMac-6 monocytic cells, again verifying their anti-inflammatory properties. Structure–activity relationship (SAR) classification models based on atom pair descriptors and physicochemical ADME parameters were developed to achieve better insight into the relationships between chemical structures of the compounds and their biological activities, and we found that there was little correlation between FPR agonist activity and inhibition of LPS-induced NF-κB activity. Indeed, Cmpd43, a well-known pyrazolone-based FPR agonist, as well as FPR1 and FPR2 peptide agonists had no effect on the LPS-induced NF-κB activity in THP1-Blue cells. Thus, some FPR agonists reported to have anti-inflammatory activity may actually mediate their effects through FPR-independent pathways, as it is suggested by our results with this series of compounds. This could explain how treatment with some agonists known to be inflammatory (i.e., FPR1 agonists) could result in anti-inflammatory effects. Further research is clearly needed to define the molecular targets of pyridazinones and structurally related compounds with anti-inflammatory activity and to define their relationships (if any) to FPR signaling events.

## 1. Introduction

Inflammation is a biological defensive mechanism in response to harmful stimuli, such as pathogens, damaged cells, or irritants, and it can last for a short period of time or it can result in a chronic condition [1,2]. Persistent inflammation can contribute to a variety of pathologies; therefore, adequate control of the inflammatory response is essential [3]. During the development of inflammation, phagocytes play a major role because they are involved in the host immune defense and are able to produce a variety of bioactive inflammatory mediators, which, if accumulated, can cause cell and tissue damage [4]. Indeed, inflammation plays a central role in gastritis, colitis, dermatitis, rheumatoid arthritis, pulmonary disease, and type II diabetes [5]. Furthermore, it has been shown that some cancers [6], Alzheimer’s disease [7], atherosclerosis, cardiovascular disease [8], and certain neurological disorders [9] are associated with persistent inflammation.

Although a wide range of compounds are available for the treatment of inflammatory diseases, it is still important to identify new and more effective drugs with less toxic collateral effects to treat both acute and chronic inflammation. Current anti-inflammatory compounds are able to reduce the production or activity of certain cytokines or their receptors (anti-cytokine therapies), while others are able to block lymphocyte trafficking into tissues, preventing the binding of monocyte–lymphocyte costimulatory molecules or reducing the number of circulating B lymphocytes. In addition, the potential of targeting several biochemical pathways and multiple enzymes involved in inflammation, including neuroinflammation, has been reported [10].

The pyridazinone structure serves as a potential scaffold for the development of novel anti-inflammatory drugs [11,12]. For example, some pyridazinone derivatives have been reported to inhibit cyclooxygenase 2 (COX2) [13,14]; thus, they belong to the group of nonsteroidal anti-inflammatory drugs (NSAID). Other pyridazinone derivatives are able to inhibit lipopolysaccharide (LPS)-induced neuroinflammation [15,16]. Recently, pyridazinone derivatives containing an indole moiety were reported as potential phosphodiesterase type 4 (PDE4) inhibitors with anti-inflammatory activity [17,18]. Elagawany et al. [19] synthetized a new inhibitor of dual specificity tyrosine phosphorylation-regulated kinase 1A (DYRK1A) and glycogen synthase kinase-3 (GSK3) based on a pyridazinone scaffold. Additionally, pyridazinone derivatives have been shown to target cell division cycle 7-related protein kinase (Cdc7) [20] and were recently shown to be potent transient receptor potential channel 5 (TrpC5) inhibitors [21]. Another area of pyridazinone development has been in the discovery of N-formyl peptide receptor (FPR) ligands that can modulate leukocyte inflammatory activities [22,23,24,25,26,27,28,29,30].

FPRs are a family of G-protein-coupled receptors (GPCR) involved in a wide range of physiological, as well as pathological processes, including inflammation, and there are three FPR isoforms in humans: FPR1, FPR2, and FPR3 [31,32,33,34]. FPR1/FPR2 agonists and antagonists have been postulated to be pro-resolving agents and potential therapeutics for treating inflammatory diseases [35,36]. Previously, we designed and synthetized a number of compounds with pyridazinone-like scaffolds in an effort to identify novel FPR ligands [22,23,24,25,26,27,28,29]. For example, we identified a mixed FPR1/FPR2 agonist, known as Compound 17b [**Cmpd17b**; N-(4-bromophenyl)-2-[5-(3-methoxybenzyl)-3-methyl-6-oxopyridazin-1(6*H*)-yl]-propanamide] [22]. **Cmpd17b** has been reported by other groups to exhibit a wide spectrum of biological activities in vivo, including endothelium-independent relaxation of mouse aortas [37] and cardioprotection from myocardial infarction injury in mice [38]. Likewise, FPR agonists with 2-pyridinone and pyridazinone scaffolds were reported to reduce pain hypersensitivity in rats with complete Freund’s adjuvant (CFA)-induced inflammatory arthritis [26,39]. Similarly, **Cmpd43** [N-(4-chlorophenyl)-N′-(5-isopropyl-1-methyl-3-oxo-2-phenyl-2,3-dihydro-1*H*-pyrazol-4-yl)urea], a pyrazolone-based FPR agonist, was found to have anti-inflammatory effects in murine ear inflammation and air-pouch models of inflammation [40,41,42], and it reduced osteoclastogenesis, joint swelling, tissue inflammation, synovitis, and cartilage damage in animal models with rheumatoid arthritis [43,44]. **Cmpd43** also reduced left ventricular (LV) anterior wall infarct scar size [45], although in another study, this compound was ineffective in reducing early cardiac necrosis and inflammation and it was unable to attenuate an LV remodeling 7 days post-ischemia reperfusion injury [38]. Recently, cardiac structure and functional improvements were observed in a mouse heart failure model following treatment with **BMS-986235/LAR-1219** (1-[(3*S*,4*R*)-4-(2,6-difluoro-4-methoxyphenyl)-2-oxopyrrolidin-3-yl]-3-phenylurea), a pyrrolidine-based FPR agonist [46].

In an effort to identify new anti-inflammatory compounds, we evaluated the anti-inflammatory activity of a library of 177 compounds consisting mostly of pyridazinone-like derivatives that were previously evaluated for FPR1/FPR2 agonist activity in order to understand if FPR1/FPR2 agonist activity was related to anti-inflammatory activity. In initial screening assays, the effects of the compounds on lipopolysaccharide (LPS)-induced NF-κB activity in THP1 monocytes/macrophages was evaluated, as NF-κB activation is an important component of inflammatory responses [47,48]. Selected compounds were further evaluated for their effects on LPS-induced interleukin 6 (IL-6) production in monocytic MonoMac-6 cells. Finally, a structure–activity relationship (SAR) classification model based on atom pair descriptors and physicochemical ADME parameters was developed to achieve better insight into the relationships between the chemical structures of the investigated compounds and their biological activity. We identified a number of pyridazinone-like compounds with anti-inflammatory activity and concluded that the pyridazinone scaffold could be useful for the development of anti-inflammatory therapeutics.

## 2. Results and Discussion

### 2.1. Screening of Compounds for Anti-Inflammatory Activity

A library of 177 compounds, which was previously evaluated for FPR1/FPR2 agonist activity (including both active FPR agonists and inactive compounds), was evaluated for the ability to inhibit LPS-induced NF-κB transcriptional activity using transfected human monocytic THP1-Blue cells [49]. THP1 monocytic cells express FPR1-3 [50,51,52]; therefore, we were able to evaluate the FPR-dependent and FPR-independent responses. These derivatives included compounds with a range of pyridazinone-like scaffolds, including pyridazinones (**1**–**120**, series A1–A4, 120 compounds), 4,5-dihydro-pyridazinones (**121**–**132**, series B, 12 compounds), indoles (**133**, **134**, series C, 2 compounds), pyridazines (**135**–**138**, series D, 4 compounds), 2-pyridinones (**139**–**148**, series E, 10 compounds), 2,6-pyrimidinediones (**149**–**154**, series F, 6 compounds), pyrazoles and pyrazolones (**155**–**164**, series G, 10 compounds), thiazol-2-ones (**165**–**173**, series H, 9 compounds), and 4 bicyclic compounds (**174**–**177**, series I) [22,23,24,25,26,27,28,29,53,54]. The structures of the compounds and the previously reported FPR1/FPR2 agonist activity, as determined by their ability to induce Ca^2+^ mobilization, are shown in Table 1. Note that the synthesis of compounds **61** and **139** and their activity at FPR1/FPR2 are reported here for the first time (see Supplementary Materials).

**Figure 1:**
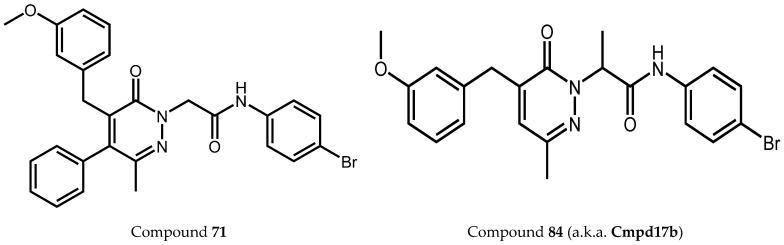
Structures of pyridazinones **71** [N-(4-bromophenyl)-2-[5-(3-methoxybenzyl)-3-methyl-6-oxo-4-phenylpyridazin-1(6*H*)-yl]-acetamide] and **84** [N-(4-bromophenyl)-2-[5-(3-methoxybenzyl)-3-methyl-6-oxopyridazin-1(6*H*)-yl]-propanamide].

**Figure 2:**
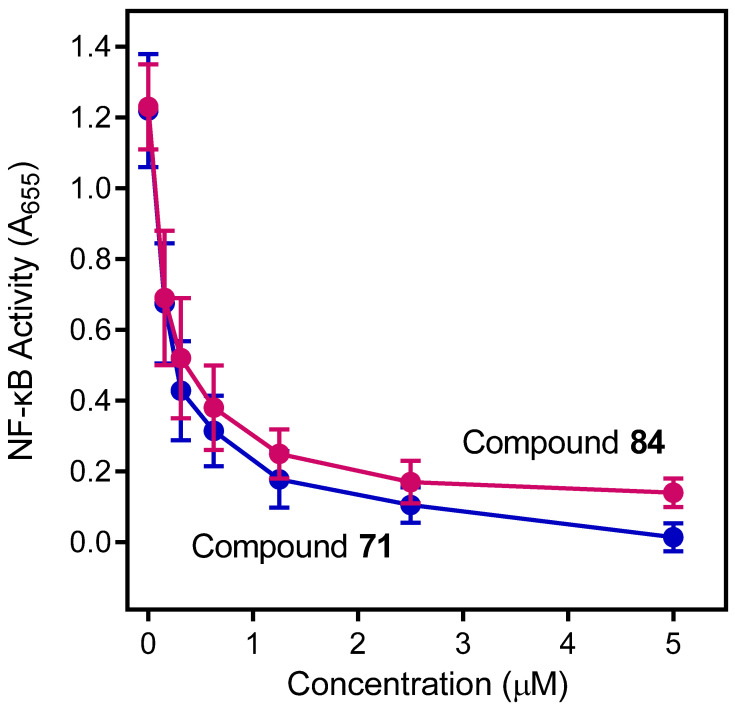
The effects of compounds **71** and **84** on LPS-induced NF-κB activation. THP1-Blue cells were pretreated with the indicated concentrations of the compounds or DMSO for 30 min, followed by an addition of 250 ng/mL LPS or buffer for 24 h. NF-κB activation was monitored by measuring secreted alkaline phosphatase activity in the cell supernatants, as described. The data are presented as the mean ± S.D. of triplicate samples from one experiment that is representative of three independent experiments.

Among the 177 compounds tested, 48 compounds inhibited the LPS-induced NF-κB activity in THP1-Blue cells (Table 2), suggesting that they could potentially be novel anti-inflammatory molecules. The chemical structures of the most potent pyridazinones (compounds **71** and **84**) are shown in Figure 1. Representative dose–response curves showing inhibition of the LPS-induced activation of NF-κB activity by these compounds are shown in Figure 2. Interestingly, 34 of these compounds were previously found to be FPR ligands with varying potencies and specificities (Table 1), whereas 14 of these NF-κB pathway inhibitors were shown previously to have no FPR activity, suggesting that they target FPR-independent anti-inflammatory pathways in THP1 cells. Nevertheless, there was not a good correlation between the FPR agonist activity and anti-inflammatory activity, as only 4 of the 17 most potent FPR agonists (EC_50_ < 1 µM at FPR1 or FPR2) inhibited the LPS-induced NF-κB activity. Consistent with this conclusion, **Cmpd43**, a documented pyrazolone-based dual FPR1/2 agonist [40,41,42] and the high-affinity FPR1 and FPR2 peptide agonists (5 nM fMLF and 5 nM WKYMVM, respectively) also did not inhibit the LPS-induced NF-κB activity in THP1-Blue cells. Indeed, we previously found that peptide agonists of FPR1/FPR2 actually enhanced NF-κB activity in THP1-Blue cells by 2–2.5 fold in comparison with a solvent control [57]. Importantly, 46 of these compounds had no cytotoxicity, whereas **66** and **158** had some cytotoxicity (IC_50_ = 22–26 μM) (Table 2).

The pyridazinone scaffold (**1**–**120**) was present in 70% of the active compounds in the anti-inflammatory assay, although this could be related to the greater number of molecules tested that had this specific structure. The chiral centers in **94**/**95**, **98**/**99**, and **102**/**103** affected the activity, even though it was not clear if there was a preferential enantiomer. Additionally, substitution of the chiral carbon led to an active compound only when small groups were present. The N-(4-bromophenyl)-acetamide group was present in almost all of the compounds tested, and all compounds with major modifications of this fragment were inactive, except for **109** and **113**. On the other hand, some modifications in this group tended to improve the anti-inflammatory activity, as exemplified by compounds **2**, **5**, and **15**, where either a shift from the para to the meta position or a replacement of the bromine with a nitro group or a fluorine atom improved activity compared to the inactive compound **1**. Furthermore, changes in the 3-methoxybenzyl chain, which is present in many of the compounds, improved the anti-inflammatory activity, as seen for **30**, where the methoxy group was removed, or for **64**, where the same methoxy group was substituted with a fluorine atom. Interestingly, the pyridazinone derivative containing just a methyl group at position 3 (compound **67**) was active.

When a para fluorine was present on the phenyl of the acetamide chain in position 1, a substitution of the methyl group with a para-phenyl-substituted group at position 5 (compounds **42**, **46**, and **47**) led to active compounds. The same result occurred even when there was an N-(4-bromophenyl)-acetamide moiety, as in compound **49** compared to the inactive compound **19**. Position 4 of the pyridazinone scaffold was mostly unsubstituted, but the insertion of an increasingly bulkier group led to an improved activity, as shown by compounds **69**, **71**, **78**, **79**, and **80**, which contain a methyl, ethyl, n-propyl, and n-butyl, respectively. Reduction of the double bond on C3–C4 was not favorable for activity (dihydro derivatives **121**–**132**), nor was the presence of a condensed ring on the pyridazinone scaffold (compounds **174**, **175**, **176**, and **177**). Likewise, compounds having an indole (**133** and **134**) or a pyridazine core (**135**–**138**) were inactive, even though the latter might be due to the lack of an N-1 acetamide chain or, moreover, a pyridinone core, with the only exception being compound **141**. Considering the small number of pyrimidin-2,6-dione derivatives analyzed (**149**–**154**, 6 compounds) and that 4 out of 6 compounds were active, this might also be the most promising scaffold to further investigate for anti-inflammatory compound development. Analysis of the 5-member ring compounds (**155**–**173**) showed that a number of these compounds were active and could be potential leads for further development. Although the molecule in this group having the best IC_50_ (compound **158**) was cytotoxic, it could also be further investigated for potential modifications that may reduce/eliminate cytotoxic effects. 

**Figure 3:**
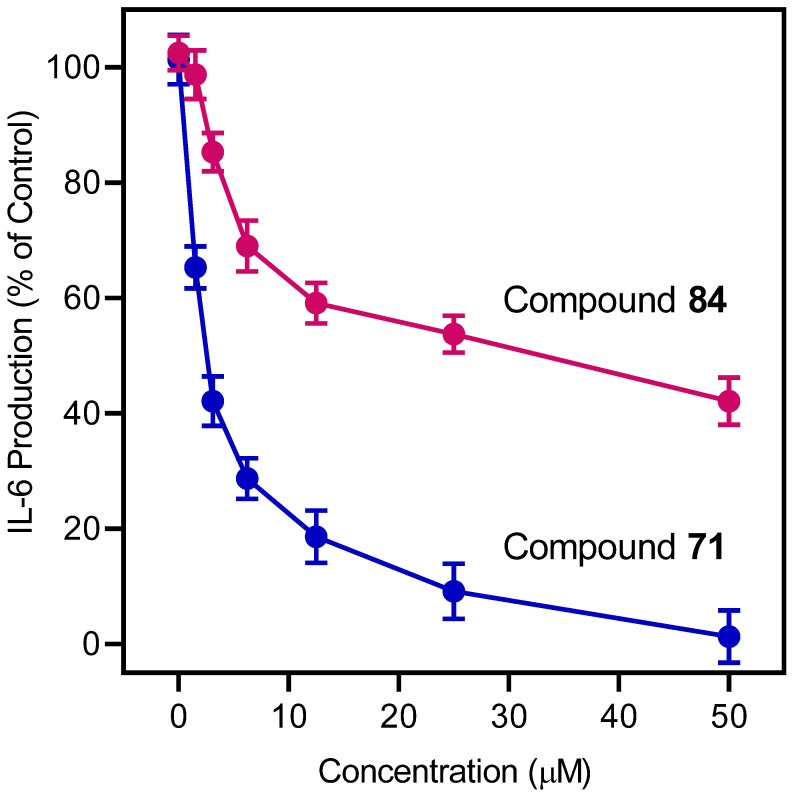
The effect of compounds **71** and **84** on the LPS-induced monocyte IL-6 production. MonoMac-6 cells were pretreated with the indicated concentrations of the test compounds or DMSO for 30 min, followed by an addition of 250 ng/mL LPS or buffer for 24 h. The production of IL-6 in the supernatants was evaluated by ELISA, as described. The data are presented as the mean ± S.D. of triplicate samples from one experiment that is representative of three independent experiments.

The most potent anti-inflammatory compounds (based on the inhibition of LPS-induced NF-κB activity in THP1-Blue cells) were also evaluated for their effect on LPS-induced IL-6 production in MonoMac-6 cells, which is also considered to be an inflammatory response (Table 3). All of these compounds, except **46**, were also found to be active in this cell-based assay system and inhibited IL-6 production. Compound **71** again had the highest activity (IC_50_ = 2.0 µM), whereas compound **84** had reduced activity (IC_50_ = 30.7 µM) compared to its effectiveness in THP1-Blue cells. Representative dose-dependent response curves showing inhibition of the LPS-induced activation of IL-6 production by the pyridazinones **71** and **84** are shown in Figure 3. The pyrimidine-2,6-dione derivatives (**150** and **153**) had an IC_50_ < 10 µM and were also potent IL-6 inhibitors, as was compound **169**, which has a 5-member ring scaffold. The only compound exhibiting cytotoxicity in MonoMac-6 cells was compound **9**. It should be noted that among the 24 non-cytotoxic inhibitors of IL-6 production, 6 structurally related pyridazinones (**2**, **38**, **42**, **46**, **47**, and **83**) were not FPR1 or FPR2 agonists (Table 1), again supporting our previous suggestion that FPR agonist activity does not correlate well with anti-inflammatory activity. Indeed, the potent peptide agonists of FPR1 and FPR2 (fMLF and WKYMVM, respectively) also did not affect the LPS-induced IL-6 production by MonoMac-6 cells.

### 2.2. Classification Tree Analysis

**Figure 4:**
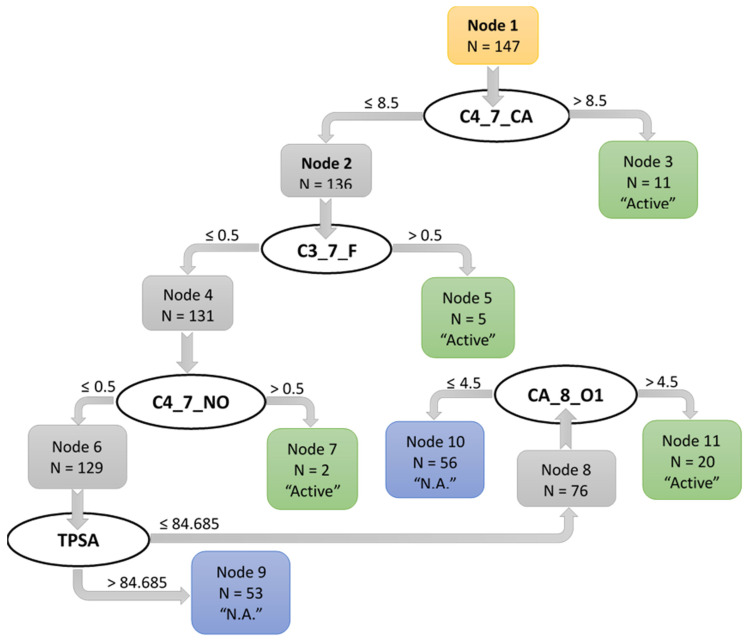
A binary classification tree reflecting the simplified SAR rules for predicting the inhibition of the NF-κB transcription activity by pyridazinones and related derivatives.

To further evaluate the relationships between the chemical structures of the investigated compounds and their anti-inflammatory activity (i.e., the inhibition of LPS-induced NF-κB activity), we performed analysis based on a binary classification tree approach. The classification tree was developed using ADME parameters and atomic pairs as 2D descriptors. A total of 147 compounds were included in the analysis, as the racemic mixtures along with the individual enantiomers corresponding to these racemates were excluded. The ADME parameters were calculated using SwissADME [59], and the selected parameters found are shown in Supplementary Table S1. The total number of different atom pairs present in at least one compound was 1029, with topological distances between atoms ranging from 1 to 22 chemical bonds. The number of occurrences of an atom pair in a molecule was considered to be the value of the corresponding 2D descriptor [60]. Atom pairs have an important advantage in SAR analysis over many other types of descriptors, since they can be easily related to certain substructures in a molecule [60,61,62]. Therefore, the results can be interpreted in terms of molecular fragments and/or functional groups. The classification tree is based on five descriptors [four atom pairs and one ADME parameter, known as topological polar surface area (tPSA)]. The misclassification matrix in graphical and tabular forms are shown in Figure 4 and Table 4, respectively. 

**Figure 5:**
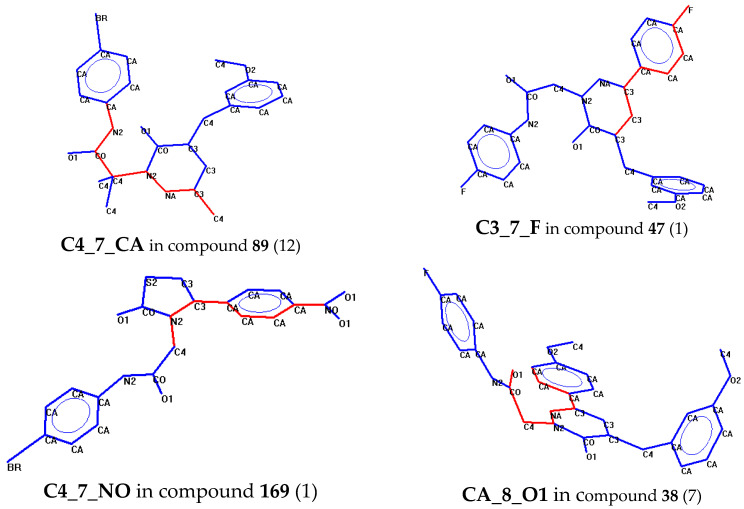
Examples of molecular structures with atom pairs corresponding to C4_7_CA, C3_7_F, C4_7_NO, and CA_8_O1 that were used for predicting the inhibition of the LPS-induced NF-κB activity. One atom pair for each structure is highlighted in red and the total occurrence of the atom pair of a given type is indicated in parentheses. The notation of the atom types is as follows: CA, aromatic carbon; C3, olefin-type or imino carbon; C4, tetrahedral sp3-hybridized carbon; CO, carbonyl carbon; NA, pyridine nitrogen; N1, nitrogen in nitro group; N2, amino-, amido-, or imino-nitrogen; O1, carbonyl or nitro oxygen; O2, two-coordinated alcohol or ether-type oxygen; BR, bromine.

According to the classification tree model, 123 of the 147 compounds evaluated (83.7%) were classified correctly, whereas 11 of 36 active compounds were erroneously classified as inactive (Supplementary Table S2). This may be due to the uneven distribution of compounds within the class, i.e., there were relatively many inactive and few active compounds. According to the logical rules defined by the classification tree, a compound was classified as active if it had at least one of the C3_7_F or C4_7_NO atom pairs or more than eight C4_7_CA atom pairs (terminal nodes 5, 7, and 3, respectively). Otherwise, the compound was recognized as active if it had a tPSA value of not greater than 84.685 Å and, at the same time, contained more than four CA_8_O1 atom pairs (terminal node 11). Examples of compounds containing the mentioned 2D descriptors are shown in Figure 5.

The atom pairs C4_7_CA were present in molecules containing simultaneously alkyl groups and aromatic fragments at a distance of several chemical bonds. The 2D descriptor C3_7_F corresponds to the presence of a fluorine-containing substituent on the phenyl or benzyl group attached to the non-aromatic pyridazinone heterocycle, while the C4_7_NO atom pair corresponds to the presence of a nitrophenyl fragment in the acetamide derivative. The condition on the last split of the tree, using the CA_8_O1 atom pair, corresponds to an arylacetamide derivative with a pyridazinone heterocycle and an aryl substituent in this heterocycle in the position opposite of the carbonyl group. This results in at least five CA_8_O1 atom pairs in the molecule.

The importance of the C3_7_F and C4_7_NO atom pairs for the NF-κB inhibitory activity correlates well with the SAR analysis, described above. Indeed, these 2D descriptors are due to fluorine and nitro substituents at certain topological distances from the acetamide and/or heterocyclic moiety (see examples in Figure 5). The topological polar surface area (tPSA) is also an important descriptor in the binary classification tree (Figure 4). Thus, 53 compounds with tPSA > 84.685 Å^2^ were recognized as inactive (terminal node 9), which agrees well with the known fact that a high number of polar groups is unfavorable for cell permeability [63,64].

The classification rules encoded in the binary tree can be generally translated into “chemical” language applied to the series of compounds that we investigated. According to the classification model, a compound is active if at least one of the following three conditions is satisfied: **Condition 1** (corresponds to node 3 of the tree)—a compound contains a pyridazinone heterocycle which has two or more substituents with sp^3^ carbon atoms (e.g., phenylacetamide fragment or benzyl group) and an aliphatic moiety with more than one sp^3^ carbon atom (e.g., isopropyl, **10**; cyclohexyl, **18**; propyl, **79**; etc.). **Condition 2** (corresponds to nodes 5 and 7 of the tree)—a compound contains a pyridazinone heterocycle that has a phenyl or benzyl substituent containing a strong acceptor (i.e., a fluorine atom, **47**, **49**, **64**; a trifluoromethyl group, **66**; or a *p*-nitro group, **169**), or a compound contains a *p*-nitroacetamide moiety (**5**). **Condition 3** (corresponds to node 11 of the tree)—a compound contains at least two C=O groups and two or more benzene rings (e.g., pyridazinones with the phenyl group in position 3 and the acetamide moiety in position 1 meet these structural criteria), and the compound has a relatively low tPSA value (≤84.685 Å^2^).

Thus, taking into account the 2D descriptors (atom pairs) in combination with the ADME physicochemical parameters made it possible to develop SAR models based on binary trees containing simple classification rules, with the possibility of interpreting the rules in terms of chemical substructures in the compounds under study.

### 2.3. Comparative Analysis

To evaluate the pairwise relationship between anti-inflammatory activity of these compounds in the cell-based assay in THP1-Blue cells and their FPR1/2 agonist activity in the Ca^2+^ mobilization assay using transfected HL-60 cells, we used the Jaccard index. This is defined in mathematical terms as the ratio of the intersection over the union for two subsets, A and B, with different activities within the investigated series of compounds [65] and as follows:

$$J\left(A,B\right)=\frac{\left|A\cap B\right|}{\left|A\cup B\right|}$$

where the numerator corresponds to the number of compounds exhibiting both biological activities, while the denominator is the number of compounds exhibiting at least one of the activities. As shown in Table 5, the relationship between FPR1 and FPR2 agonist activities is tight, with a Jaccard index of 0.831, while the inhibition of the LPS-induced NF-κB activity is only weakly related to the compound’s FPR1/FPR2 agonist activity.

**Figure 6:**
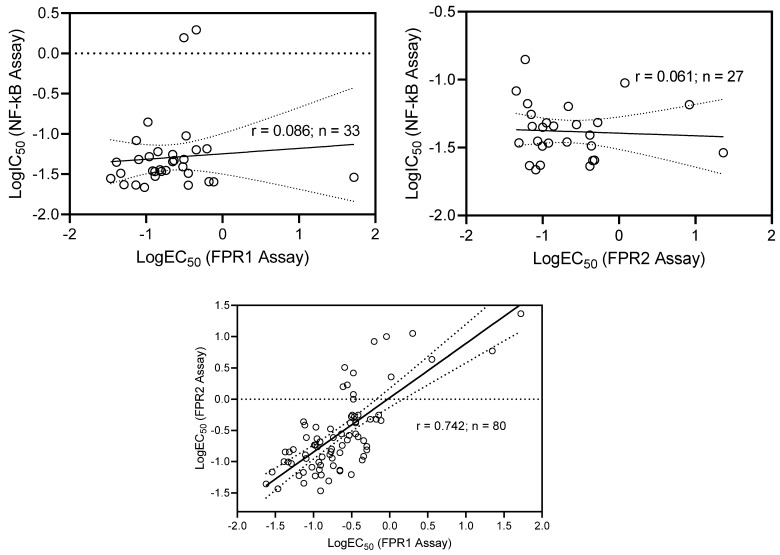
Correlation of NF-κB inhibitory activity and FPR1/FPR2 agonist activity for compounds manifesting both type of activities. The activities are represented as a logarithm (logIC_50_ or logEC_50_).

No correlation was found when plotting the logarithms of IC_50_ and EC_50_ values for the NF-κB inhibitory and FPR1/FPR2 activation assays, respectively, and for compounds active in both types of assays (r = 0.086 for NF-κB vs. FPR1 and r=0.061 for NF-κB vs. FPR2). However, a relatively strong linear correlation (r = 0.742, n = 80) was obtained for agonists of both FPR1 and FPR2 (Figure 6).

Thus, the calculated Jaccard indices and Pearson correlation coefficients indicate that there is not a significant relationship between NF-κB inhibitory activity (i.e., anti-inflammatory activity) and FPR1/FPR2 agonist activity. Indeed, this result is also consistent with the lack of anti-inflammatory activity observed for the FPR1 and FPR2 peptide agonists.

**Figure 7:**
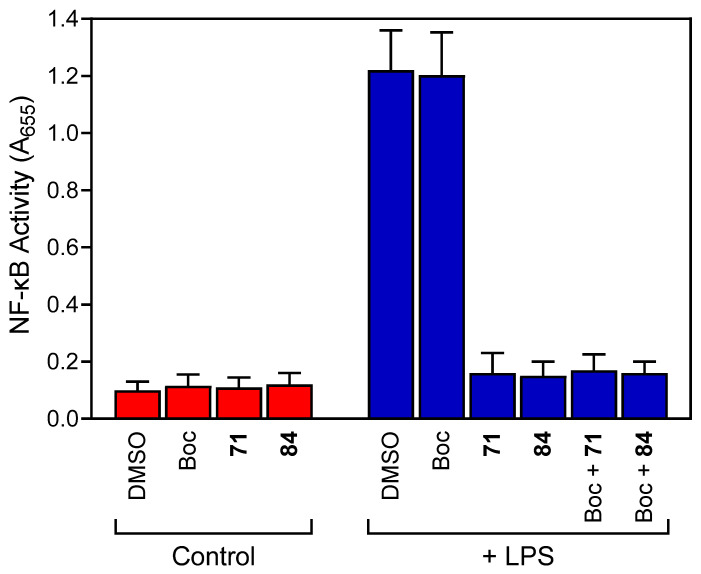
The effect of Boc-MLF on the anti-inflammatory activity of compounds **71** and **84**. THP1-Blue cells were pretreated for 15 min with buffer or 25 μM Boc-MLF, followed by treatment of 30 min with DMSO or 5 µM of compounds **71** and **84**. The cells were then incubated for 24 h with media (control) or 250 ng/mL LPS, as indicated. NF-κB activation was monitored by measuring the secreted alkaline phosphatase activity spectrophotometrically in the cell supernatants (A_655_), as described. The data are presented as the mean ± S.D. of triplicate samples from one experiment that is representative of three independent experiments.

Among the pyridazinones and related derivatives evaluated for anti-inflammatory activity were 98 FPR1/FPR2 agonists and 79 compounds with no FPR agonist activity. Our analysis revealed that 46 of these compounds were not cytotoxic and exhibited anti-inflammatory activity, as determined by their ability to inhibit the LPS-induced NF-κB activity in human THP1-Blue monocytic cells. The most potent of these compounds also inhibited monocyte/macrophage IL-6 production, further supporting their anti-inflammatory potential. While the anti-inflammatory compounds identified included both FPR agonists and non-agonists, the calculated Jaccard indices and Pearson correlation coefficients indicated that there was not a significant relationship between the anti-inflammatory activity and FPR1/FPR2 agonist activity. This is an interesting finding, as several groups have reported that FPR agonists can exhibit anti-inflammatory activity in in vivo inflammatory models. For example, compound **84** (a.k.a. FPR1 agonist **Cmpd17b**) was reported to induce endothelium-independent relaxation in mouse aortas and had a cardioprotection effect in myocardial infarction injury in mice [22,37,38]. We also found that this compound had anti-inflammatory activity using in vitro assays. In addition, an evaluation of the effect of an FPR1 antagonist (Boc-MLF) on the ability of compounds **84** and **71** (both are FPR1-specific agonists) showed that their anti-inflammatory effects on LPS-induced NF-κB activation were independent of FPR1, since blocking FPR1 had no effect on their inhibitor activity in LPS-treated THP1 cells (Figure 7). 

Thus, the question is whether some of the FPR agonists reported to have anti-inflammatory activity actually mediate their effects through FPR-independent pathways, as is suggested by our results with these series of compounds. This could explain how treatment with some agonists known to be inflammatory (i.e., FPR1 agonists) could result in anti-inflammatory effects. At the very least, it should be considered in FPR studies that some FPR agonists may mediate their effects in vivo through both FPR-dependent and FPR-independent pathways, possibly to include homologous down-regulation of FPR in a process called functional antagonism [66]. As another example, FPR agonists **69**, **71**, and **73** were all reported previously to reduce pain hypersensitivity in rats with CFA-induced inflammatory arthritis [26]. Interestingly, compounds **69** and **71** inhibited LPS-induced NF-κB activity, and compound **71** inhibited LPS-induced IL-6 production, whereas **73** was inactive. Thus, FPR-dependent and FPR-independent anti-inflammatory effects should be considered when making conclusions regarding the therapeutic potential of these compounds. In comparison, the anti-inflammatory effects of the pyrazolone-based FPR1/FPR2 dual agonist (i.e., **Cmpd43**) have been reported in several murine models of inflammation [40,41,42,43,44]; however, this compound did not inhibit an LPS-induced NF-kB activity, suggesting that it is likely acting in vivo through signaling pathways not involving NF-kB. Nevertheless, additional targets should also be considered when evaluating the anti-inflammatory effects of this class of compounds. For example, several pyridazinones structurally related to compounds **1–120** were reported to be positive allosteric modulators of the α7 nicotinic acetylcholine receptor (nAChR) [67]. It is known that such positive allosteric modulators can act on α7 nAChRs producing anti-nociceptive activity [68,69]. The nAChRs are expressed not only by neurons but also by macrophages and other immune cells involved in inflammation [70,71]. In these cells, stimulation of the nAChR by specific agonists suppresses the release of pro-inflammatory cytokines and could have a therapeutic potential for the alleviation of rheumatoid arthritis [72]. Another potential target of pyridazine compounds could be monoamine oxidase (MAO), and pyridazinones structurally related to the compounds evaluated here were reported as MAO-A inhibitors [73,74]. MAO inhibitors have anti-inflammatory effects in the CNS and a variety of non-CNS tissues [75]. Moreover, MAO-A inhibitors could be used for the reprogramming of tumor-associated macrophages to improve cancer immunotherapy [76].

## 3. Materials and Methods

### 3.1. Materials

DMSO, N-formyl-Met-Leu-Phe (fMLF), and Trp-Lys-Tyr-Met-Val-Met (WKYMVM) were purchased from Sigma-Aldrich Chemical Co. (St. Louis, MO, USA). **Cmpd43** (TC-FPR 43) and Boc-MLF were purchased from Tocris Biosciences (Bristol, UK). Fluo-4AM was purchased from Invitrogen (Carlsbad, CA, USA). Roswell Park Memorial Institute (RPMI) 1640 medium was purchased from HyClone Laboratories (Logan, UT, USA). Fetal calf serum and fetal bovine serum (FBS) were purchased from ATCC (Manassas, VA, USA). Hanks’ balanced salt solution was purchased from Life Technologies (Grand Island, NY, USA). HBSS without Ca^2+^ and Mg^2+^ was designated as HBSS^−^. HBSS containing 1.3 mM CaCl_2_ and 1.0 mM MgSO_4_ was designated as HBSS^+^.

### 3.2. Compounds

Our studies included 175 compounds that were previously synthesized and characterized at NEUROFARBA, Pharmaceutical and Nutraceutical Section, University of Florence, Italy. We previously evaluated the FPR1/FPR2 agonist activity of these compounds by evaluating their ability to induce Ca^2+^ mobilization in FPR-transfected HL60 cells and human neutrophils [22,23,24,25,26,27,28,29,53,54]. Compounds **61** and **139** were synthesized as described (see Supplementary Materials). All test compounds were dissolved in DMSO at stock concentrations of 10 mM and stored at −20 °C. For primary screening assays (analysis of NF-κB activation, IL-6 production, and cytotoxicity), the following final concentrations of the compounds were used: 3.125, 6.25, 12.5, 25, and 50 µM; the final concentration of DMSO was 1% in all samples. The most potent compounds were retested at final concentrations in the submicromolar and low micromolar range (0.1–5.0 µM).

### 3.3. Cell Culture

All cells were cultured at 37 °C in a humidified atmosphere containing 5% CO_2_. The THP1-Blue cells (InvivoGen, San Diego, CA, USA) were cultured in an RPMI 1640 medium (Mediatech Inc., Herndon, VA, USA) supplemented with 10% (*v*/*v*) fetal bovine serum (FBS), 100 μg/mL streptomycin, 100 U/mL penicillin, 100 μg/mL phleomycin (Zeocin), and 10 μg/mL blasticidin S. Human monocyte-macrophage MonoMac-6 cells (Deutsche Sammlung von Mikroorganismen und Zellkulturen GmbH, Braunschweig, Germany) were grown in an RPMI 1640 medium supplemented with 10% (*v*/*v*) FBS, 10 μg/mL bovine insulin, 100 μg/mL streptomycin, and 100 U/mL penicillin. 

### 3.4. Analysis of NF-κB Activation

The activation of NF-κB was measured using an alkaline phosphatase reporter assay in human monocytic THP1-Blue cells stably transfected with a secreted embryonic alkaline phosphatase gene that is under the control of a promoter inducible by the NF-κB. The THP1-Blue cells (2 × 10^5^ cells/well) were pretreated with the test compound or DMSO for 30 min, followed by an addition of 250 ng/mL LPS for 24 h. Alkaline phosphatase activity was determined in cell supernatants using a QUANTI-Blue mix (InvivoGen) and measured at 655 nm and compared with positive control samples (LPS). For active compounds, the concentrations of the inhibitors that caused a 50% inhibition of the NF-κB reporter activity (IC_50_) were calculated.

### 3.5. IL-6 Analysis

A human IL-6 ELISA kit (BD Biosciences, San Jose, CA, USA) was used to measure IL-6 production. MonoMac-6 cells were plated in 96-well plates at a density of 2 × 10^5^ cells/well in a culture medium supplemented with 3% (*v*/*v*) endotoxin-free FBS. The cells were pretreated with the test compound or DMSO for 30 min, followed by addition of 250 ng/mL LPS for 24 h. The IC_50_ values for IL-6 production were calculated by plotting the percentage inhibition against the logarithm of the inhibitor concentration (at least five points).

### 3.6. Cytotoxicity Assay

The cytotoxicity was analyzed with a CellTiter-Glo Luminescent Cell Viability Assay Kit from Promega (Madison, WI, USA), according to the manufacturer’s protocol. The luminescent ATP assays are based on a luciferase enzymatic reaction which uses ATP from viable cells to generate photons. For this assay, the cells were incubated with the compounds under investigation for 24 h. After treatment, the cells were equilibrated to room temperature for 30 min, luciferase + luciferin substrate was added, and the samples were analyzed with a Fluoroscan Ascent FL (Thermo Fisher Scientific, Waltham, MA, USA). The IC_50_ values were calculated by plotting the percentage inhibition against the logarithm of the inhibitor concentration (at least five points).

### 3.7. Ca^2+^ Mobilization Assay

Changes in the intracellular Ca^2+^ [Ca^2+^]_i_ were measured with a FlexStation II scanning fluorometer (Molecular Devices). The cells, which were suspended in Hank’s balanced salt solution without Ca^2+^ and Mg^2+^ but with 10 mM HEPES (HBSS^−^), were loaded with 1.25 μg/mL Fluo-4 AM dye and incubated for 30 min in the dark at 37 °C. After the dye loading, the cells were washed with HBSS^−^ containing 10 mM HEPES, resuspended in HBSS^+^ containing Ca^2+^, Mg^2+^, and 10 mM HEPES (HBSS^+^), and aliquoted into the wells of black microtiter plates (2 × 10^5^ cells/well). For the evaluation of the direct agonist activity, the compounds of interest were added from a source plate containing dilutions of the test compounds in HBSS^+^ and changes in the fluorescence were monitored (λ_ex_ = 485 nm, λ_em_ = 538 nm) every 5 s for 240 s at room temperature after the automated addition of the compounds. The maximum change in the fluorescence during the first 3 min, expressed in arbitrary units over the baseline, was used to determine a response. The responses for the FPR1 agonists were normalized to the response induced by 5 nM fMLF for FPR1-HL60 cells or 5 nM WKYMVM for FPR2-HL60 cells, which were assigned a value of 100%. A curve fitting (5–6 points) and a calculation of median effective inhibitory concentrations (IC50) were performed by nonlinear regression analysis of the dose–response curves generated using Prism 8 (GraphPad Software, Inc., San Diego, CA, USA).

### 3.8. Molecular Modeling

For SAR classification analysis, we used an atom pair representation of molecular structures with each atom pair denoted as T1_D_T2, where T1 and T2 are the types of atoms in the pair and D represents the topological distance or number of bonds in the shortest path between these atoms in a structural formula. In our investigation, T1 and T2 were defined with symbolic codes used in HyperChem, Version 7 (Hypercube, Inc., Waterloo, ON, Canada) for an atom type representation within an MM+ force field [60,61,62]. For example, CA, CO, and C3 codes were used for sp^2^-hybridized aromatic, carbonyl, and pyrazole carbon atoms, respectively. This approach allows easy generation of atom pairs directly from the output file containing the molecular structure (HIN file) built by HyperChem.

A total of 147 compounds were included in the analysis. The racemic mixtures along with individual enantiomers corresponding to these racemates were excluded, as they are indistinguishable in the atom pair 2D representation of the molecular structures. All 1029 unique atom pairs possible for non-hydrogen atoms in the 147 investigated compounds were generated. This 147×1029 data matrix was automatically built by our CHAIN program based on HIN files created in HyperChem. By convention, a matrix element at the intersection of the i^th^ row and j^th^ column was equal to the j^th^ atom pair occurrence in the i^th^ molecule. The data matrix obtained in this way for the 147 compounds was supplemented with columns corresponding to the physicochemical ADME parameters calculated with the use of a SwissADME online tool (http://www.swissadme.ch, accessed on 1 August 2021) [59]. 

The total matrix containing both atom pair and ADME descriptors was used for the construction of SAR rules with the use of a binary classification tree methodology [77] with STATISTICA 6.0 using discriminant-based univariate splits with estimated prior probabilities and equal misclassification costs for classes.

## 4. Conclusions

Previously, we identified a number of FPR1/FPR2 agonists and found that many of the active compounds had a pyridazinone core structure [22,23,24,25,26,27,28,29]. In an effort to identify novel anti-inflammatory compounds, we evaluated a library of 177 pyridazinone-like compounds and related scaffolds. Although the pyridazinone scaffold was present in ~70% of the compounds that inhibited the LPS-induced NF-κB activity in a cell-based anti-inflammatory assay, there was not a good correlation between the FPR1/FPR2 agonist activity and the anti-inflammatory activity. We suggest that some of these compounds could mediate their anti-inflammatory effects through FPR-independent pathways, which may explain why treatment with some agonists that are known to be inflammatory (i.e., FPR1 agonists) results in anti-inflammatory effects. We developed a SAR classification model based on atom pair descriptors and physicochemical ADME parameters and found that the C4_7_CA, C3_7_F, CA_8_O1, and C4_7_NO atom pairs, together with the topological polar surface area (tPSA), a physicochemical descriptor, were important. This classification model could provide a relatively simple approach for *de novo* design of anti-inflammatory compounds with pyridazinone-like scaffolds. Further research is clearly needed to define the molecular targets of pyridazinones and structurally related compounds with anti-inflammatory activity and to define their relationships (if any) to FPR signaling events.

## Figures and Tables

**Table 1:** Chemical structure and FPR1/FPR2 agonist activity of derivatives used for the evaluation of anti-inflammatory activity in human THP1-Blue cells.

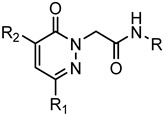 Series A1: Pyridazinones							
Compd.	R	R_1_	R_2_	FPR1	FPR2		Ref.
				**EC_50_ (µM)**			
**1**	p-Br-Ph	CH_3_	m-(OCH_3_)-Bn	3.4	3.8		[22]
**2**	m-Br-Ph	CH_3_	m-(OCH_3_)-Bn	N.A.	N.A.^a^		[22]
**3**	o-Br-Ph	CH_3_	m-(OCH_3_)-Bn	N.A.	N.A.		[22]
**4**	p-Cl-Ph	CH_3_	m-(OCH_3_)-Bn	2.6	4.0		[22]
**5**	p- NO_2_-Ph	CH_3_	m-(OCH_3_)-Bn	10.5	12.3		[22]
**6**	p,m-(OCH_3_)-Ph	CH_3_	m-(OCH_3_)-Bn	15.5	16.8		[22]
**7**	p-CF_3_-Ph	CH_3_	m-(OCH_3_)-Bn	5.7	8.8		[22]
**8**	p-Br-Ph	Ph	m-(OCH_3_)-Bn	9.0	4.3		[23]
**9**	p-Br-Ph	CH_3_	CH_2_-3-thienyl	4.5	14.1		[23]
**10**	p-Br-Ph	iPr	m-(OCH_3_)-Bn	4.5	7.2		[23]
**11**	p-Br-Ph	CH_3_	CH_2_-2-thienyl	8.1	11.4		[23]
**12**	p-Br-Ph	H	m-(OCH_3_)-Bn	6.1	7.7		[23]
**13**	p-Br-Ph	CH_3_	CH_2_-1-naphtyl	13.8	N.A.		[23]
**14**	p-(tBu)-Ph	CH_3_	m-(OCH_3_)-Bn	N.A.	N.A.		[22]
**15**	p-F-Ph	CH_3_	m-(OCH_3_)-Bn	7.6	N.A.		[22]
**16**	Ph	CH_3_	m-(OCH_3_)-Bn	N.A.	N.A.		[22]
**17**	p-CN-Ph	CH_3_	m-(OCH_3_)-Bn	N.A.	N.A.		[22]
**18**	p-Br-Ph	C_6_H_11_	m-(OCH_3_)-Bn	10.8	N.A.		[23]
**19**	p-Br-Ph	CH_3_	p-(OCH_3_)-Ph	11.2	N.A.		[23]
**20**	p-I-Ph	Et	m-(OCH_3_)-Bn	4.2	5.5		[23]
**21**	p-I-Ph	CH_3_	p-(SCH_3_)-Bn	2.3	9.4		[28]
**22**	p-I-Ph	CH_3_	m,m-(OCH_3_)-Bn	7.6	N.A.		[23]
**23**	p-I-Ph	CH_3_	m-Cl-Bn	9.5	16.9		[23]
**24**	p-(SCH_3_)-Ph	CH_3_	m-(OCH_3_)-Bn	2.2	8.2		[28]
**25**	p-I-Ph	CH_3_	H	N.A.	N.A.		[23]
**26**	p-Br-Ph	CH_3_	NH_2_	8.1	29.4		[23]
**27**	p-Br-Ph	CH_3_	NHCO-p-Br-Ph	N.A.	N.A.		[23]
**28**	p-F-Ph	C_6_H_11_	H	N.A.	N.A.		[53]
**29**	p-F-Ph	CH_3_	Bn	N.A.	N.A.		[23]
**30**	p-Br-Ph	CH_3_	Bn	5.5	11.6		[23]
**31**	5-benzo[d][1,3]dioxole	C_6_H_11_	H	N.A.	N.A.		[53]
**32**	5-benzo[d][1,3]dioxole	CH_3_	Bn	6.9	N.A.		[23]
**33**	5-benzo[d][1,3]dioxole	Ph	m-(OCH_3_)-Bn	N.A.	N.A.		[23]
**34**	p-F-Ph	Ph	m-(OCH_3_)-Bn	N.A.	N.A.		[23]
**35**	5-benzo[d][1,3]dioxole	2-thienyl	m-(OCH_3_)-Bn	N.A.	N.A.		[23]
**36**	p-F-Ph	2-thienyl	m-(OCH_3_)-Bn	N.A.	N.A.		[23]
**37**	p-Br-Ph	2-thienyl	p-(OCH_3_)-Bn	N.A.	N.A.		[23]
**38**	p-F-Ph	p-(OCH_3_)-Ph	m-(OCH_3_)-Bn	N.A.	N.A.		[23]
**39**	5-benzo[d][1,3]dioxole	p-(OCH_3_)-Ph	m-(OCH_3_)-Bn	N.A.	N.A.		[23]
**40**	p-Br-Ph	p-(OCH_3_)-Ph	p-(OCH_3_)-Bn	N.A.	N.A.		[23]
**41**	p-Br-Ph	p-Cl-Ph	p-(OCH_3_)-Bn	N.A.	N.A.		[23]
**42**	p-F-Ph	p-Cl-Ph	m-(OCH_3_)-Bn	N.A.	N.A.		[23]
**43**	5-benzo[d][1,3]dioxole	p-Cl-Ph	m-(OCH_3_)-Bn	N.A.	N.A.		[23]
**44**	p-Br-Ph	p-CH_3_-Ph	p-(OCH_3_)-Bn	N.A.	N.A.		[23]
**45**	5-benzo[d][1,3]dioxole	p-CH_3_-Ph	m-(OCH_3_)-Bn	N.A.	N.A.		[23]
**46**	p-F-Ph	p-CH_3_-Ph	m-(OCH_3_)-Bn	N.A.	N.A.		[23]
**47**	p-F-Ph	p-F-Ph	m-(OCH_3_)-Bn	N.A.	N.A.		[23]
**48**	5-benzo[d][1,3]dioxole	p-F-Ph	m-(OCH_3_)-Bn	N.A.	N.A.		[23]
**49**	p-Br-Ph	p-F-Ph	p-(OCH_3_)-Bn	N.A.	N.A.		[23]
**50**	p-Br-Ph	CH_3_	N(p-(OCH_3_)-Ph)_2_	N.A.	N.A.		[53]
**51**	p-Br-Ph	CH_3_	NH-p-(OCH_3_)-Ph	12.8	7.8		[23]
**52**	p-Br-Ph	CH_3_	NHCO-m-(OCH_3_)-Ph	9.3	2.8		[23]
**53**	p-Br-Ph	CH_3_	CO-m-(OCH_3_)-Ph	3.0	1.0		[23]
**54**	p-Br-Ph	Bn	p-(OCH_3_)-Bn	N.A.	N.A.		[23]
**55**	p-Br-Ph	CH_3_	m-Br-Bn	N.A.	N.A.		[23]
**56**	p-Br-Ph	CH_3_	m,m-(OCH_3_)-Bn	N.A.	N.A.		[23]
**57**	p-F-Ph	Ph	CH_3_	N.A.	N.A.		[27]
**58**	p-Br-Ph	CH_3_	p-(SCH_3_)-Bn	N.A.	N.A.		[28]
**59**	p-Br-Ph	CH_3_	CH_2_-3-furyl	5.8	6.3		[23]
**60**	p-Br-Ph	CH_3_	CH_2_-3-Pyridyl	9.3	2.8		[23]
**61**	p-Br-Ph	CH_3_	p-Bn-CONH-(p-Br-Ph)	N.A.	N.A.		Suppl.^a^
**62**	p-Br-Ph	CH_3_	p-(CONH_2_)-Bn	29.3	27.2		[23]
**63**	p-Br-Ph	CH_3_	p-CN-Bn	N.A.	N.A.		[23]
**64**	p-Br-Ph	CH_3_	m-F-Bn	6.6	N.A.		[23]
**65**	p-Br-Ph	CH_3_	m-Cl-Bn	10.5	N.A.		[23]
**66**	p-Br-Ph	CH_3_	p-CF_3_-Bn	N.A.	N.A.		[23]
**67**	p-Br-Ph	Ph	CH_3_	21.5	10.1		[27]
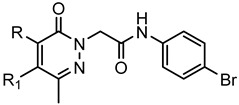 **Series A2:** **Pyridazinones**							
**Compd.**	**R**	**R_1_**	**FPR1**	**FPR2**			**Ref.**
			**EC_50_ (µM)**				
**68**	H	CH_3_	5.7	0.51			[25]
**69**	m-(OCH_3_)-Bn	CH_3_	0.019	0.043			[25]
**70**	H	Ph	N.A.	0.15			[25]
**71**	m-(OCH_3_)-Bn	Ph	2.2	N.A.			[25]
**72**	NH_2_	CO(CH)_2_-N(CH_3_)_2_	5.1	5.7			[24]
**73**	NH-m-(OCH_3_)-Ph	Ac	0.045	0.17			[24]
**74**	NH_2_	m-Pirazolyl	8.4	13.5			[24]
**75**	NH_2_	1-CH_3_-3-Pyrazolyl	2.9	1.9			[24]
**76**	NH-m-(OCH_3_)-Ph	1-CH_3_-3-Pyrazolyl	3.6	0.59			[24]
**77**	NH-p-(OCH_3_)-Ph	1-CH_3_-3-Pyrazolyl	4.0	0.035			[24]
**78**	m-(OCH_3_)-Bn	Et	3.2	1.9			[25]
**79**	m-(OCH_3_)-Bn	nPr	2.2	4.6			[25]
**80**	m-(OCH_3_)-Bn	nBu	N.A.	15.7			[25]
**81**	NH-m-(OCH_3_)-Ph	nBu	3.6	4.5			[24]
**82**	NH-m-(OCH_3_)-Ph	H	0.24	9.6			[24]
**83**	NH-m-(OCH_3_)-Ph	nPr	N.A.	N.A.			[24]
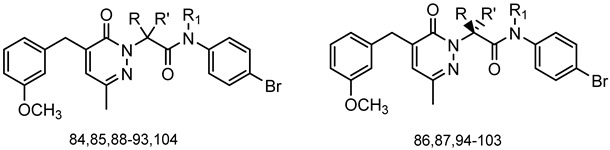 **Series A3: Pyridazinones**							
**Compd.**	**R**	**R’**	**R_1_**	**FPR1**	**FPR2**		**Ref.**
				**EC_50_ (µM)**			
**84**	CH_3_	H	H	3.2	N.A.		[22]
**85**	H	H	CH_3_	N.A.	N.A.		[22]
**86**	H	CH_3_	H	8.5	10.2		[55]
**87**	CH_3_	H	H	3.2	16.1		[55]
**88**	Et	H	H	1.3	2.2		[55]
**89**	CH_3_	CH_3_	H	6.3	20.4		[55]
**90**	Et	CH_3_	H	1.5	2.1		[55]
**91**	nPr	H	H	2.8	3.6		[55]
**92**	iPr	H	H	2.0	6.5		[55]
**93**	nBu	H	H	1.1	0.1		[55]
**94**	H	Et	H	2.8	2.3		[55]
**95**	Et	H	H	13.4	22.2		[55]
**96**	H	iPr	H	9.4	5.4		[55]
**97**	iPr	H	H	N.A.	N.A.		[55]
**98**	H	nPr	H	3.0	0.84		[55]
**99**	nPr	H	H	N.A.	N.A.		[55]
**100**	H	nBu	H	0.5	0.089		[55]
**101**	nBu	H	H	20.8	7.0		[55]
**102**	CH_3_	Et	H	4.5	13.7		[55]
**103**	Et	CH_3_	H	7.0	N.A.		[55]
**104**	Ph	H	H	3.1	1.8		[55]
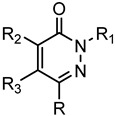 **Series A4: Pyridazinones**							
**Compd.**	**R**	**R_1_**	**R_2_**	**R_3_**	**FPR1**	**FPR2**	**Ref.**
					**EC_50_ (µM)**		
**105**	H	p-(OCH_3_)-Bn	Cl	p-(OBu)-Ph	N.A.	N.A.	[53]
**106**	CH_3_	Ph	NH-p-(OBu)-Ph	H	N.A.	N.A.	[22]
**107**	H	m-(OCH_3_)-Bn	Cl	p-(OBu)-Ph	N.A.	N.A.	[53]
**108**	H	m-(OCH_3_)-Bn	p-(OBu)-Ph	OCH_3_	N.A.	N.A.	[53]
**109**	H	p-(OCH_3_)-Bn	p-(OBu)-Ph	OCH_3_	N.A.	N.A.	[53]
**110**	CH_3_	CH_2_CO-N-(CH_3_)-Pip	m-(OCH_3_)-Bn	H	N.A.	N.A.	[22]
**111**	CH_3_	(CH_2_)_2_CONH-p-Br-Ph	m-(OCH_3_)-Bn	H	9.7	5.4	[22]
**112**	CH_3_	CH_2_COO-p-Br-Ph	m-(OCH_3_)-Bn	H	N.A.	N.A.	[22]
**113**	CH_3_	(CH_2_)_2_NHCONH-p-Br-Ph	m-(OCH_3_)-Bn	H	N.A.	N.A.	[22]
**114**	CH_3_	(CH_2_)_2_NHCO-p-Br-Ph	m-(OCH_3_)-Bn	H	N.A.	N.A.	[22]
**115**	CH_3_	m-(OCH_3_)-Bn	NHCONH-p-Br-Ph	H	N.A.	N.A.	[22]
**116**	CH_3_	CH_2_NHCO-p-Br-Ph	m-(OCH_3_)-Bn	H	N.A.	N.A.	[22]
**117**	CH_3_	m-(OCH_3_)-Bn	NHCO-p-Br-Ph	H	N.A.	N.A.	[22]
**118**	CH_3_	CH_2_NHCONH-p- Br-Ph	m-(OCH_3_)-Bn	H	N.A.	N.A.	[22]
**119**	CH_3_	CH_2_-CS-NH-p- Br-Ph	m-(OCH_3_)-Bn	H	N.A.	N.A.	[28]
**120**	CH_3_	CH_2_CONH-p-Br-Ph	NH-p-(OCH_3_)-Ph	Ac	13.5	1.7	[23]
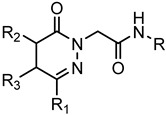 **Series B: 4,5-Dihydro-pyridazinones**							
**Compd.**	**R**	**R_1_**	**R_2_**	**R_3_**	**FPR1**	**FPR2**	**Ref.**
					**EC_50_ (µM)**		
**121**	p-I-Ph	CH_3_	H	H	N.A.	N.A.	[23]
**122**	p-F-Ph	Ph	CH_3_	H	N.A.	N.A.	[27]
**123**	5-benzo[d][1,3]dioxole	Ph	CH_3_	H	N.A.	N.A.	[27]
**124**	p-Br-Ph	Ph	CH_3_	H	19.5	10.7	[27]
**125**	p-Br-Ph	Ph	CH_3_	H	24.4	10.0	[27]
**126**	p-F-Ph	Ph	CH_3_	H	N.A.	N.A.	[27]
**127**	p-F-Ph	Ph	CH_3_	H	N.A.	N.A.	[27]
**128**	5-benzo[d][1,3]dioxole	Ph	CH_3_	H	N.A.	N.A.	[27]
**129**	5-benzo[d][1,3]dioxole	Ph	CH_3_	H	N.A.	N.A.	[27]
**130**	p-Br-Ph	Ph	CH_3_	H	23.5	7.0	[27]
**131**	p-Br-Ph	CH_3_	H	CH_3_	13.0	2.6	[25]
**132**	p-Br-Ph	CH_3_	H	Ph	4.1	0.63	[25]
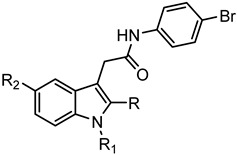 **C: Indoles**							
**Compd.**	**R**	**R_1_**	**R_2_**	**FPR1**	**FPR2**		**Ref.**
				**EC_50_ (µM)**			
**133**	CH_3_	CO-p-Cl-Ph	OCH_3_	N.A.	N.A.		[54]
**134**	H	m-(OCH_3_)-Bn	H	N.A.	N.A.		[56]
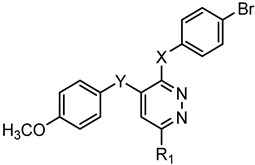 **Series D: Pyridazines**							
**Compd.**	**X**	**Y**	**R_1_**	**FPR1**	**FPR2**		**Ref.**
				**EC_50_ (µM)**			
**135**	NHCONH	O	Ph	N.A.	N.A.		[28]
**136**	NHCO	O	Ph	N.A.	N.A.		[28]
**137**	NHCH_2_CONH	O	Ph	N.A.	N.A.		[28]
**138**	SCH_2_CONH	CH_2_	CH_3_	N.A.	N.A.		[28]
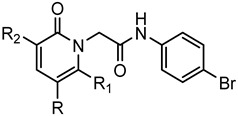 **Series E: 2-Pyridinones**							
**Compd.**	**R**	**R_1_**	**R_2_**	**FPR1**	**FPR2**		**Ref.**
				**EC_50_ (µM)**			
**139**	4-Pyridyl	H	NH_2_	N.A.	N.A.		Suppl.^a^
**140**	m,p-(OCH_3_)-Ph	CH_3_	CN	33.2	0.60		[26]
**141**	m-(OCH_3_)-Ph	CH_3_	CN	1.6	0.12		[26]
**142**	COOEt	CH_3_	CN	3.0	0.38		[26]
**143**	p-(OCH_3_)-Ph	CH_3_	CN	1.6	0.12		[26]
**144**	COOEt	CH_3_	CONH-m-(OCH_3_)-Ph	0.4	28.9		[26]
**145**	COOEt	CH_3_	CONH-p-(OCH_3_)-Ph	7.9	16.4		[26]
**146**	4-Pyridyl	CH_3_	CONH-m-(OCH_3_)-Ph	1.4	1.8		[26]
**147**	CONH-m-(OCH_3_)-Ph	CH_3_	CN	3.9	0.31		[26]
**148**	CONH-p-(OCH_3_)-Ph	CH_3_	CN	0.96	0.44		[26]
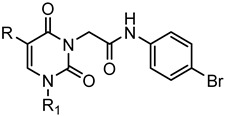 **Series F: 2,6-Pyrimidinediones**							
**Compd.**	**R**	**R_1_**	**FPR1**	**FPR2**			**Ref.**
			**EC_50_ (µM)**				
**149**	H	p-CH_3_-Ph	13.6	14.9			[26]
**150**	H	m-(OCH_3_)-Ph	4.3	3.6			[26]
**151**	Ph	p-CH_3_-Ph	N.A.	N.A.			[26]
**152**	H	nPr	5.5	4.1			[26]
**153**	H	Ph	12.5	8.9			[26]
**154**	m-Ph(OCH_3_)	nPr	3.3	2.4			[26]
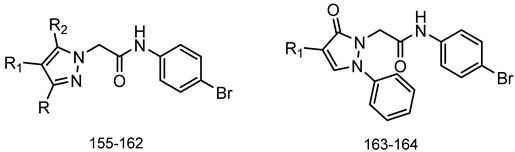 **Series G: Pyrazoles and Pyrazolones**							
**Compd.**	**R**	**R_1_**	**R_2_**	**FPR1**	**FPR2**		**Ref.**
				**EC_50_ (µM)**			
**155**	H	CN	NH-m-(OCH_3_)-Ph	N.A.	N.A.		[29]
**156**	H	CN	NH-p-(OCH_3_)-Ph	N.A.	N.A.		[29]
**157**	Ph	CH_3_	NH_2_	N.A.	N.A.		[29]
**158**	Ph	CH_3_	NH-m-(OCH_3_)-Ph	N.A.	N.A.		[29]
**159**	Ph	CH_3_	NH-p-(OCH_3_)-Ph	N.A.	N.A.		[29]
**160**	m-(OCH_3_)-Ph	CN	CH_3_	18.4	6.1		[29]
**161**	p-(OCH_3_)-Ph	CN	CH_3_	N.A.	N.A.		[29]
**162**	H	H	NH-m-(OCH_3_)-Ph	13.2	23.4		[29]
**163**	-	H	-	N.A.	23.1		[29]
**164**	-	NH-m-(OCH_3_)-Ph	-	N.A.	N.A.		[29]
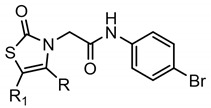 **Series H: Thiazol-2-ones**							
**Compd.**	**R**	**R_1_**	**FPR1**	**FPR2**			**Ref.**
			**EC_50_ (µM)**				
**165**	Ph	H	1.8	2.1			[25]
**166**	m-(OCH_3_)-Ph	H	0.28	0.23			[25]
**167**	p-(OCH_3_)-Ph	H	2.6	1.8			[25]
**168**	p-Cl-Ph	H	2.8	2.4			[25]
**169**	p-NO_2_-Ph	H	9.1	N.A.			[25]
**170**	m-Cl-Ph	H	6.0	3.0			[25]
**171**	p-NH_2_-Ph	H	34.9	14.7			[25]
**172**	CH_3_	Ac	8.9	5.8			[25]
**173**	CH_3_	H	12.4	4.1			[25]
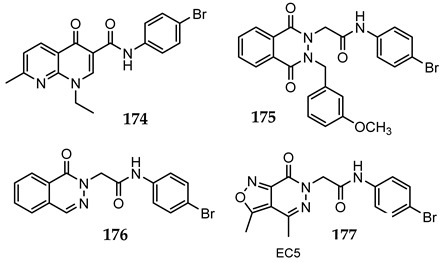 **Series I: Bicyclic** **compounds**							
**Compd.**		**FPR1**		**FPR2**		**Ref.**	
		**EC_50_ (µM)**					
**174**		N.A.		N.A.		[56]	
**175**		N.A.		N.A.		[56]	
**176**		N.A.		N.A.		[56]	
**177**		18.4		6.4		[24]	

^a^ Suppl.: see Supplementary Materials. N.A.: No activity was observed at the highest concentration tested (50 µM). Pip, piperazine; Ph, phenyl; Bn, benzyl; iPr, iso-propyl; tBu, tert-butyl; Et, ethyl; nPr, normal-propyl; nBu, normal-butyl; Ac, acetyl.

**Table 2:** The effects of the test compounds and FPR peptide agonists on the LPS-induced NF-κB activity in human THP1-Blue cells, cytotoxicity, and Ca^2+^ mobilization in FPR1/FPR2-transfected HL-60 cells.

Compd.	Inhibition of NF-κB Activity	Cytotoxicity	Ca^2+^ Mobilization	
			FPR1 ^a^	FPR2 ^a^
	IC_50_ (µM) in THP1-Blue Cells		EC_50_ (µM) in HL-60 Cells	
**2**	44.5 ± 2.5	N.T.	N.A.	N.A.
**5**	46.1 ± 1.0	N.T.	10.5	12.3
**9**	18.0 ± 0.7	N.T.	4.5	14.1
**10**	22.0 ± 1.7	N.T.	4.5	7.2
**15**	33.6 ± 2.1	N.T.	7.6	N.A.
**23**	7.1 ± 2.0	N.T.	9.5	16.9
**30**	28.4 ± 5.7	N.T.	5.5	11.6
**38**	19.6 ± 4.3	N.T.	N.A.	N.A.
**42**	22.5 ± 2.3	N.T.	N.A.	N.A.
**46**	8.2 ± 1.2	N.T.	N.A.	N.A.
**47**	3.4 ± 0.2	N.T.	N.A.	N.A.
**49**	34.5 ± 2.1	N.T.	N.A.	N.A.
**50**	32.8 ± 0.7	N.T.	N.A.	N.A.
**64**	27.9 ± 2.1	N.T.	6.6	N.A.
**66**	31.5 ± 2.2	26.4 ± 4.7	N.A.	N.A.
**67**	30.9 ± 2.3	N.T.	21.5	10.1
**69**	34.5 ± 2.6	N.T.	0.019	0.043
**71**	0.5 ± 0.1	N.T.	2.2	N.A.
**78**	20.7 ± 0.7	N.T.	3.2	1.9
**79**	15.7 ± 1.4	N.T.	2.2	4.6
**80**	15.0 ± 1.5	N.T.	N.A.	15.7
**83**	14.6 ± 1.4	N.T.	N.A.	N.A.
**84**	0.6 ± 0.1	N.T.	3.2	N.A.
**88**	39.4 ± 4.2	N.T.	1.3	2.2
**89**	29.2 ± 1.0	N.T.	6.3	20.4
**90**	39.2 ± 6.7	N.T.	1.5	2.1
**94**	30.8 ± 6.7	N.T.	2.8	2.3
**95**	12.1 ± 2.4	N.T.	13.4	22.2
**98**	10.6 ± 2.3	N.T.	3.0	0.84
**102**	22.1 ± 1.6	N.T.	4.5	13.7
**103**	16.6 ± 0.6	N.T.	7.0	N.A.
**109**	31.3 ± 1.6	N.T.	N.A.	N.A.
**113**	37.0 ± 0.4	N.T.	N.A.	N.A.
**122**	46.3 ± 2.4	N.T.	N.A.	N.A.
**124**	42.7 ± 0.3	N.T.	19.5	10.7
**125**	22.4 ± 0.7	N.T.	24.4	10.0
**141**	15.3 ± 4.9	N.T.	1.6	0.12
**149**	43.0 ± 3.9	N.T.	13.6	14.9
**150**	21.4 ± 3.5	N.T.	4.3	3.6
**153**	20.8 ± 0.5	N.T.	12.5	8.9
**154**	25.6 ± 2.3	N.T.	3.3	2.4
**156**	25.0 ± 0.4	N.T.	N.A.	N.A.
**158**	8.8 ± 1.1	22.4 ± 5.2	N.A.	N.A.
**160**	28.8 ± 2.0	N.T.	8.2	4.8
**164**	35.7 ± 1.9	N.T.	29	N.A.
**168**	43.4 ± 4.2	N.T.	2.8	2.4
**169**	19.2 ± 0.1	N.T.	9.1	N.A.
**Cmpd43**	N.A.	N.T.	0.065	0.022
***f*** **MLF**	N.A. ^b^	N.T.	0.01	
**WKYMVM**	N.A. ^b^	N.T.		0.001

^a^ The agonist activity at FPR1/FPR2 is from Table 1. The reported agonist activity of the dual FPR1/FPR agonist **Cmpd43** is from [42]. The FPR agonist activities of fMLF and WKYMVM are reported in [58]. N.T.: No cytotoxicity was observed at the highest concentration tested (50 µM). N.A.: No activity was observed at the highest concentration tested (50 µM). ^b^ fMLF and WKYMVM were tested at 5 nM.

**Table 3:** The effects of selected compounds and FPR peptide agonists on the LPS-induced production of IL-6 in human MonoMac-6 cells, cytotoxicity, and Ca^2+^ mobilization in FPR1/FPR2-transfected HL-60 cells.

Compd.	Inhibition of IL-6 Production	Cytotoxicity	Ca^2+^ Mobilization	
			FPR1 ^a^	FPR2 ^a^
	IC_50_ (µM) in MonoMac-6 Cells		EC_50_ (µM) in HL-60 Cells	
**2**	30.7 ± 3.6	N.T.	N.A.	N.A.
**9**	27.5 ± 1.6	19.0 ± 3.6	4.5	14.1
**10**	8.7 ± 1.6	N.T.	4.5	7.2
**23**	13.1 ± 0.3	N.T.	9.5	16.9
**38**	20.3 ± 1.8	N.T.	N.A.	N.A.
**42**	35.6 ± 3.1	N.T.	N.A.	N.A.
**46**	N.A.^b^	N.T.	N.A.	N.A.
**47**	18.8 ± 2.9	N.T.	N.A.	N.A.
**71**	2.0 ± 0.3	N.T.	2.2	N.A.
**78**	17.0 ± 0.4	N.T.	3.2	1.9
**79**	13.7 ± 1.4	N.T.	2.2	4.6
**80**	33.5 ± 2.4	N.T.	N.A.	15.7
**83**	15.2 ± 0.2	N.T.	N.A.	N.A.
**84**	30.7 ± 1.8	N.T.	3.2	N.A.
**89**	27.9 ± 2.2	N.T.	6.3	20.4
**94**	14.3 ± 2.0	N.T.	2.8	2.3
**95**	9.8 ± 1.0	N.T.	13.4	22.2
**98**	5.6 ± 0.3	N.T.	3.0	0.84
**102**	19.0 ± 3.1	N.T.	4.5	13.7
**103**	7.3 ± 1.2	N.T.	7.0	N.A.
**125**	7.2 ± 0.03	N.T.	24.4	10.0
**141**	24.5 ± 0.8	N.T.	1.6	0.12
**150**	4.0 ± 1.2	N.T.	4.3	3.6
**153**	10.7 ± 0.1	N.T.	12.5	8.9
**154**	2.6 ± 0.3	N.T.	3.3	2.4
**169**	3.2 ± 0.4	N.T.	9.1	N.A.
***f*** **MLF**	N.A. ^b^	N.T. ^d^	0.01	
**WKYMVM**	N.A. ^b^	N.T. ^d^		0.001

^a^ Agonist activity at FPR1/FPR2, as reported previously (see Table 1). N.T.: No cytotoxicity was observed at the highest concentration tested (50 µM). N.A.: No activity was observed at the highest concentration tested (50 µM). ^b^ fMLF and WKYMVM were tested at 5 nM.

**Table 4:** Misclassification matrices for the binary classification trees obtained for the NF-κB inhibitory activity of the test compounds.

		Observed	
		Active	Non-Active
		36	111
**Predicted**	**Active**	25	13
	**Non-active**	11	98

**Table 5:** Jaccard indices for different pairs of biological activities.

Activity Classes	Number of Compounds		J (A,B)
	|A ∪ B|	|A ∩ B|	
FPR1 vs. FPR2	77	64	64/77 = 0.831
FPR1 vs. NF-κB	89	21	21/89 = 0.236
FPR2 vs. NF-κB	85	18	18/85 = 0.212

## Data Availability

The data presented in this study are available on request from the corresponding author.

## Supplementary Materials

The following supporting information can be downloaded at: https://www.mdpi.com/article/10.3390/molecules27123749/s1. Synthesis of compounds **61** and **139.** Table S1: Selected ADME parameters of the investigated compounds calculated with SwissADME web tool. Table S2: Experimentally observed and predicted classes for inhibition of NF-κB activity for the pyridazinones and related derivatives in according to classification tree analysis [78].

## Sample Availability

Samples of the compounds are not available from the authors.

## References

[1] Medzhitov R. (2008). Origin and physiological roles of inflammation. Nature.

[2] Chen L., Deng H., Cui H., Fang J., Zuo Z., Deng J., Li Y., Wang X., Zhao L. (2018). Inflammatory responses and inflammation-associated diseases in organs. Oncotarget.

[3] Nathan C., Ding A. (2010). Nonresolving inflammation. Cell.

[4] Fujiwara N., Kobayashi K. (2005). Macrophages in inflammation. Curr. Drug. Targets. Inflamm. Allergy.

[5] Rojas M., Woods C.R., Mora A.L., Xu J., Brigham K.L. (2005). Endotoxin-induced lung injury in mice: Structural, functional, and biochemical responses. Am. J. Physiol. Lung. Cell. Mol. Physiol..

[6] Munn L.L. (2017). Cancer and inflammation. Wiley Interdiscip. Rev. Syst. Biol. Med..

[7] Wyss-Coray T., Rogers J. (2012). Inflammation in Alzheimer Disease—A Brief Review of the Basic Science and Clinical Literature. Cold Spring Harb. Perspect. Med..

[8] Lee Y.-W., Kim P.H., Lee W.-H., Hirani A.A. (2010). Interleukin-4, Oxidative Stress, Vascular Inflammation and Atherosclerosis. Biomol. Ther..

[9] Urrutia P., Aguirre P., Esparza A., Tapia V., Mena N.P., Arredondo M., González-Billault C., Núñez M.T. (2013). Inflammation alters the expression of DMT1, FPN1 and hepcidin, and it causes iron accumulation in central nervous system cells. J. Neurochem..

[10] Zhou J., Jiang X., He S., Jiang H., Feng F., Liu W., Qu W., Sun H. (2019). Rational Design of Multitarget-Directed Ligands: Strategies and Emerging Paradigms. J. Med. Chem..

[11] Saini M., Mehta D.K., Das R., Saini G. (2016). Recent Advances in Anti-inflammatory Potential of Pyridazinone Derivatives. Mini-Reviews Med. Chem..

[12] Pau A., Catto M., Pinna G., Frau S., Murineddu G., Asproni B., Curzu M.M., Pisani L., Leonetti F., Loza M.I. (2015). Multitarget-Directed Tricyclic Pyridazinones as G Protein-Coupled Receptor Ligands and Cholinesterase Inhibitors. ChemMedChem.

[13] Ahmed E.M., Kassab A.E., El-Malah A.A., Hassan M.S. (2019). Synthesis and biological evaluation of pyridazinone derivatives as selective COX-2 inhibitors and potential anti-inflammatory agents. Eur. J. Med. Chem..

[14] Peregrym K., Szczukowski L., Wiatrak B., Potyrak K., Czyznikowska Z., Swiatek P. (2021). In Vitro and In Silico Evaluation of New 1,3,4-Oxadiazole Derivatives of Pyrrolo[3,4-d]pyridazinone as Promising Cyclooxygenase Inhibitors. Int. J. Mol. Sci..

[15] Wakulik K., Wiatrak B., Szczukowski Ł., Bodetko D., Szandruk-Bender M., Dobosz A., Świątek P., Gąsiorowski K. (2020). Effect of Novel Pyrrolo[3,4-d]pyridazinone Derivatives on Lipopolysaccharide-Induced Neuroinflammation. Int. J. Mol. Sci..

[16] Potyrak K., Wiatrak B., Krzyżak E., Szczukowski Ł., Świątek P., Szeląg A. (2021). Effect of pyrrolo[3,4-d]pyridazinone derivatives in neuroinflammation induced by preincubation with lipopolysaccharide or coculturing with microglia-like cells. Biomed. Pharmacother..

[17] Allart-Simon I., Moniot A., Bisi N., Ponce-Vargas M., Audonnet S., Laronze-Cochard M., Sapi J., Hénon E., Velard F., Gérard S. (2021). Pyridazinone derivatives as potential anti-inflammatory agents: Synthesis and biological evaluation as PDE4 inhibitors. RSC Med. Chem..

[18] Barberot C., Moniot A., Allart-Simon I., Malleret L., Yegorova T., Laronze-Cochard M., Bentaher A., Médebielle M., Bouillon J.-P., Hénon E. (2018). Synthesis and biological evaluation of pyridazinone derivatives as potential anti-inflammatory agents. Eur. J. Med. Chem..

[19] Elagawany M., Ibrahim M.A., Ahmed H.E.A., El Etrawy A.A., Ghiaty A., Abdel-Samii Z.K., El-Feky S.A., Bajorath J. (2013). Design, synthesis, and molecular modelling of pyridazinone and phthalazinone derivatives as protein kinases inhibitors. Bioorganic Med. Chem. Lett..

[20] Woods K.W., Lai C., Miyashiro J.M., Tong Y., Florjancic A.S., Han E.K., Soni N., Shi Y., Lasko L., Leverson J.D. (2012). Aminopyrimidinone Cdc7 Kinase Inhibitors. Bioorg. Med. Chem. Lett..

[21] Zhang Z., Chen L., Tian H., Liu M., Jiang S., Shen J., Wang K., Cao Z. (2022). Discovery of pyridazinone analogs as potent transient receptor potential canonical channel 5 inhibitors. Bioorg. Med. Chem. Lett..

[22] Cilibrizzi A., Quinn M.T., Kirpotina L.N., Schepetkin I.A., Holderness J., Ye R.D., Rabiet M.-J., Biancalani C., Cesari N., Graziano A. (2009). 6-Methyl-2,4-Disubstituted Pyridazin-3(2H)-ones: A Novel Class of Small-Molecule Agonists for Formyl Peptide Receptors. J. Med. Chem..

[23] Giovannoni M.P., Schepetkin I.A., Cilibrizzi A., Crocetti L., Khlebnikov A.I., Dahlgren C., Graziano A., Dal Piaz V., Kirpotina L.N., Zerbinati S. (2013). Further studies on 2-arylacetamide pyridazin-3(2H)-ones: Design, synthesis and evaluation of 4,6-disubstituted analogs as formyl peptide receptors (FPRs) agonists. Eur. J. Med. Chem..

[24] Vergelli C., Schepetkin I.A., Ciciani G., Cilibrizzi A., Crocetti L., Giovannoni M.P., Guerrini G., Iacovone A., Kirpotina L.N., Khlebnikov A.I. (2016). 2-Arylacetamido-4-phenylamino-5-substituted pyridazinones as formyl peptide receptors agonists. Bioorganic Med. Chem..

[25] Vergelli C., Schepetkin I.A., Ciciani G., Cilibrizzi A., Crocetti L., Giovannoni M.P., Guerrini G., Iacovone A., Kirpotina L.N., Ye R.D. (2017). Synthesis of Five- and Six-Membered*N*-Phenylacetamido Substituted Heterocycles as Formyl Peptide Receptor Agonists. Drug Dev. Res..

[26] Crocetti L., Vergelli C., Guerrini G., Cantini N., Kirpotina L.N., Schepetkin I.A., Quinn M.T., Parisio C., Mannelli L.D.C., Ghelardini C. (2020). Novel formyl peptide receptor (FPR) agonists with pyridinone and pyrimidindione scaffolds that are potentially useful for the treatment of rheumatoid arthritis. Bioorganic Chem..

[27] Cilibrizzi A., Crocetti L., Giovannoni M.P., Graziano A., Vergelli C., Bartolucci G., Soldani G., Quinn M.T., Schepetkin I.A., Faggi C. (2013). Synthesis, HPLC enantioresolution, and X-ray analysis of a new series of C5-methyl pyridazines as N-formyl peptide eceptor (FPR) agonists. Chirality.

[28] Crocetti L., Vergelli C., Cilibrizzi A., Graziano A., Khlebnikov A.I., Kirpotina L.N., Schepetkin I.A., Quinn M.T., Giovannoni M.P. (2013). Synthesis and Pharmacological Evaluation of New Pyridazin-Based Thioderivatives as Formyl Peptide Receptor (FPR) Agonists. Drug Dev. Res..

[29] Vergelli C., Khlebnikov A.I., Crocetti L., Guerrini G., Cantini N., Kirpotina L.N., Schepetkin I.A., Cilibrizzi A., Quinn M.T., Rossi P. (2021). Synthesis, biological evaluation, molecular modeling, and structural analysis of new pyrazole and pyrazolone derivatives as N-formyl peptide receptors (FPRs) agonists. Chem. Biol. Drug. Des..

[30] Deora G.S., Qin C.X., Vecchio E.A., Debono A.J., Priebbenow D.L., Brady R.M., Beveridge J., Teguh S.C., Deo M., May L.T. (2019). Substituted Pyridazin-3(2*H*)-ones as Highly Potent and Biased Formyl Peptide Receptor Agonists. J. Med. Chem..

[31] Ye R.D., Boulay F., Wang J.M., Dahlgren C., Gerard C., Parmentier M., Serhan C.N., Murphy P.M. (2009). International Union of Basic and Clinical Pharmacology. LXXIII. Nomenclature for the formyl peptide receptor (FPR) family. Pharmacol. Rev..

[32] Migeotte I., Communi D., Parmentier M. (2006). Formyl peptide receptors: A promiscuous subfamily of G protein-coupled receptors controlling immune responses. Cytokine Growth Factor Rev..

[33] Tylek K., Trojan E., Regulska M., Lacivita E., Leopoldo M., Basta-Kaim A. (2021). Formyl peptide receptor 2, as an important target for ligands triggering the inflammatory response regulation: A link to brain pathology. Pharmacol. Rep..

[34] Trojan E., Bryniarska N., Leśkiewicz M., Regulska M., Chamera K., Szuster-Głuszczak M., Leopoldo M., Lacivita E., Basta-Kaim A. (2020). The Contribution of Formyl Peptide Receptor Dysfunction to the Course of Neuroinflammation: A Potential Role in the Brain Pathology. Curr. Neuropharmacol..

[35] Maciuszek M., Cacace A., Brennan E., Godson C., Chapman T.M. (2021). Recent advances in the design and development of formyl peptide receptor 2 (FPR2/ALX) agonists as pro-resolving agents with diverse therapeutic potential. Eur. J. Med. Chem..

[36] Lee H.Y., Lee M., Bae Y. (2017). Formyl Peptide Receptors in Cellular Differentiation and Inflammatory Diseases. J. Cell. Biochem..

[37] Marshall S.A., Qin C.X., Jelinic M., O’Sullivan K., Deo M., Walsh J., Li M., Parry L.J., Ritchie R.H., Leo C.H. (2020). The Novel Small-molecule Annexin-A1 Mimetic, Compound 17b, Elicits Vasoprotective Actions in Streptozotocin-induced Diabetic Mice. Int. J. Mol. Sci..

[38] Qin C., May L.T., Li R., Cao N., Rosli S., Deo M., Alexander A.E., Horlock D., Bourke J., Yang Y.H. (2017). Small-molecule-biased formyl peptide receptor agonist compound 17b protects against myocardial ischaemia-reperfusion injury in mice. Nat. Commun..

[39] Crocetti L., Vergelli C., Guerrini G., Giovannoni M.P., Kirpotina L.N., Khlebnikov A.I., Ghelardini C., Mannelli L.D.C., Lucarini E., Schepetkin I.A. (2021). Pyridinone Derivatives as Interesting Formyl Peptide Receptor (FPR) Agonists for the Treatment of Rheumatoid Arthritis. Molecules.

[40] Bürli R.W., Xu H., Zou X., Muller K., Golden J., Frohn M., Adlam M., Plant M.H., Wong M., McElvain M. (2006). Potent hFPRL1 (ALXR) agonists as potential anti-inflammatory agents. Bioorganic Med. Chem. Lett..

[41] Dufton N., Hannon R., Brancaleone V., Dalli J., Patel H.B., Gray M., D’Acquisto F., Buckingham J.C., Perretti M., Flower R.J. (2010). Anti-Inflammatory Role of the Murine Formyl-Peptide Receptor 2: Ligand-Specific Effects on Leukocyte Responses and Experimental Inflammation. J. Immunol..

[42] Sogawa Y., Ohyama T., Maeda H., Hirahara K. (2011). Inhibition of neutrophil migration in mice by mouse formyl peptide receptors 1 and 2 dual agonist: Indication of cross-desensitization in vivo. Immunology.

[43] Odobasic D., Jia Y., Kao W., Fan H., Wei X., Gu R., Ngo D., Kitching A.R., Holdsworth S.R., Morand E. (2018). Formyl peptide receptor activation inhibits the expansion of effector T cells and synovial fibroblasts and attenuates joint injury in models of rheumatoid arthritis. Int. Immunopharmacol..

[44] Kao W., Gu R., Jia Y., Wei X., Fan H., Harris J., Zhang Z., Quinn J., Morand E., Yang Y.H. (2014). A formyl peptide receptor agonist suppresses inflammation and bone damage in arthritis. J. Cereb. Blood Flow Metab..

[45] García R.A., Ito B.R., Lupisella J., Carson N.A., Hsu M.-Y., Fernando G., Heroux M., Bouvier M., Dierks E., Kick E. (2019). Preservation of Post-Infarction Cardiac Structure and Function via Long-Term Oral Formyl Peptide Receptor Agonist Treatment. JACC: Basic Transl. Sci..

[46] Asahina Y., Wurtz N.R., Arakawa K., Carson N., Fujii K., Fukuchi K., Garcia R., Hsu M.Y., Ishiyama J., Ito B. (2020). Discovery of BMS-986235/LAR-1219: A Potent Formyl Peptide Receptor 2 (FPR2) Selective Agonist for the Prevention of Heart Failure. J. Med. Chem..

[47] Mazgaeen L., Gurung P. (2020). Recent Advances in Lipopolysaccharide Recognition Systems. Int. J. Mol. Sci..

[48] Barnes P.J., Karin M. (1997). Nuclear factor-κB: A pivotal transcription factor in chronic inflammatory diseases. N. Engl. J. Med..

[49] Schepetkin I.A., Kirpotina L.N., Khlebnikov A.I., Hanks T.S., Kochetkova I., Pascual D.W., Jutila M.A., Quinn M.T. (2012). Identification and Characterization of a Novel Class of c-Jun N-terminal Kinase Inhibitors. Mol. Pharmacol..

[50] Chen Y.-C., Chang Y.-P., Hsiao C.-C., Wu C.-C., Wang Y.-H., Chao T.-Y., Leung S.-Y., Fang W.-F., Lee C.-P., Wang T.-Y. (2020). Blood M2a monocyte polarization and increased formyl peptide receptor 1 expression are associated with progression from latent tuberculosis infection to active pulmonary tuberculosis disease. Int. J. Infect. Dis..

[51] Chen Y.C., Su M.C., Chin C.H., Lin I.C., Hsu P.Y., Liou C.W., Huang K.T., Wang T.Y., Lin Y.Y., Zheng Y.X. (2019). Formyl peptide receptor 1 up-regulation and formyl peptide receptor 2/3 down-regulation of blood immune cells along with defective lipoxin A4/resolvin D1 production in obstructive sleep apnea patients. PLoS ONE.

[52] Maddox J.F., Hachicha M., Takano T., Petasis N., Fokin V.V., Serhan C.N. (1997). Lipoxin A4 Stable Analogs Are Potent Mimetics That Stimulate Human Monocytes and THP-1 Cells via a G-protein-linked Lipoxin A4 Receptor. J. Biol. Chem..

[53] Floresta G., Crocetti L., Giovannoni M.P., Biagini P., Cilibrizzi A. (2020). Repurposing strategies on pyridazinone-based series by pharmacophore- and structure-driven screening. J. Enzym. Inhib. Med. Chem..

[54] Linari G., Spanò R. (1973). Substituted anilides of 1-(p-chlorobenzoyl)-5-methoxy-2-methyl-indole-3-acetic acid. Arzneimittelforschung.

[55] Cilibrizzi A., Schepetkin I.A., Bartolucci G., Crocetti L., Piaz V.D., Giovannoni M.P., Graziano A., Kirpotina L.N., Quinn M.T., Vergelli C. (2012). Synthesis, enantioresolution, and activity profile of chiral 6-methyl-2,4-disubstituted pyridazin-3(2H)-ones as potent N-formyl peptide receptor agonists. Bioorganic Med. Chem..

[56] Cilibrizzi A., Floresta G., Abbate V., Giovannoni M.P. (2019). iVS analysis to evaluate the impact of scaffold diversity in the binding to cellular targets relevant in cancer. J. Enzym. Inhib. Med. Chem..

[57] Schepetkin I.A., Kirpotina L.N., Tian J., Khlebnikov A.I., Ye R.D., Quinn M.T. (2008). Identification of Novel Formyl Peptide Receptor-Like 1 Agonists That Induce Macrophage Tumor Necrosis Factor α Production. Mol. Pharmacol..

[58] Schepetkin I.A., Kirpotina L.N., Khlebnikov A.I., Leopoldo M., Lucente E., Lacivita E., De Giorgio P., Quinn M.T. (2013). 3-(1H-indol-3-yl)-2-[3-(4-nitrophenyl)ureido]propanamide enantiomers with human formyl-peptide receptor agonist activity: Molecular modeling of chiral recognition by FPR2. Biochem. Pharmacol..

[59] Daina A., Michielin O., Zoete V. (2017). SwissADME: A free web tool to evaluate pharmacokinetics, drug-likeness and medicinal chemistry friendliness of small molecules. Sci. Rep..

[60] Khlebnikov A.I., Schepetkin I.A., Quinn M.T. (2010). Computational structure–activity relationship analysis of small-molecule agonists for human formyl peptide receptors. Eur. J. Med. Chem..

[61] Khlebnikov A.I., Schepetkin I.A., Quinn M.T. (2008). Structure–activity relationship analysis of N-benzoylpyrazoles for elastase inhibitory activity: A simplified approach using atom pair descriptors. Bioorganic Med. Chem..

[62] Khlebnikov A.I., Schepetkin I.A., Kirpotina L.N., Quinn M.T. (2008). Computational structure–activity relationship analysis of non-peptide inducers of macrophage tumor necrosis factor-α production. Bioorganic Med. Chem..

[63] Giner B., Lafuente C., Lapena D., Errazquin D., Lomba L. (2020). QSAR study for predicting the ecotoxicity of NADES towards Aliivibrio fischeri. Exploring the use of mixing rules. Ecotox. Env. Safe.

[64] Matsson P., Kihlberg J. (2017). How big is too big for cell permeability?. J. Med. Chem..

[65] Chung N.C., Miasojedow B., Startek M., Gambin A. (2019). Jaccard/Tanimoto similarity test and estimation methods for biological presence-absence data. BMC Bioinform..

[66] Stama M.L., Lacivita E., Kirpotina L.N., Niso M., Perrone R., Schepetkin I.A., Quinn M.T., Leopoldo M. (2017). Functional N -Formyl Peptide Receptor 2 (FPR2) Antagonists Based on the Ureidopropanamide Scaffold Have Potential To Protect Against Inflammation-Associated Oxidative Stress. ChemMedChem.

[67] Capelli A.M., Castelletti L., Salvagno C., Oliosi B., Di Lenarda E., Virginio C., Lightfoot A., Kew J.N., Teague S. (2010). Identification of novel α7 nAChR positive allosteric modulators with the use of pharmacophore in silico screening methods. Bioorganic Med. Chem. Lett..

[68] Papke R.L., Bagdas D., Kulkarni A.R., Gould T., AlSharari S.D., Thakur G.A., Damaj M.I. (2015). The analgesic-like properties of the α7 nAChR silent agonist NS6740 is associated with non-conducting conformations of the receptor. Neuropharmacology.

[69] Williams D.K., Wang J., Papke R.L. (2011). Positive allosteric modulators as an approach to nicotinic acetylcholine receptor-targeted therapeutics: Advantages and limitations. Biochem. Pharmacol..

[70] Tan Y., Chu Z., Shan H., Zhangsun D., Zhu X., Luo S. (2022). Inflammation regulation via an agonist and antagonists of α7 nicotinic acetylcholine receptors in RAW264.7 macrophages. Mar. Drugs.

[71] Richter K., Papke R.L., Stokes C., Roy D.C., Espinosa E.S., Wolf P.M.K., Hecker A., Liese J., Singh V.K., Padberg W. (2022). Comparison of the anti-inflammatory properties of two nicotinic acetylcholine receptor ligands, phosphocholine and pCF3-diEPP. Front. Cell. Neurosci..

[72] van Maanen M.A., Vervoordeldonk M.J., Tak P.P. (2009). The cholinergic anti-inflammatory pathway: Towards innovative treatment of rheumatoid arthritis. Nat. Rev. Rheumatol..

[73] Khattab S.N., Bekhit A.A., El-Faham A., El Massry A.M., Amer A. (2008). Synthesis of Some Pyridazinylacetic Acid Derivatives as a Novel Class of Monoamine Oxidase-A Inhibitors. Chem. Pharm. Bull..

[74] Özdemir Z., Alagöz M.A., Uslu H., Karakurt A., Erikci A., Ucar G., Uysal M. (2020). Synthesis, molecular modelling and biological activity of some pyridazinone derivatives as selective human monoamine oxidase-B inhibitors. Pharmacol. Rep..

[75] Ostadkarampour M., Putnins E.E. (2021). Monoamine Oxidase Inhibitors: A Review of Their Anti-Inflammatory Therapeutic Potential and Mechanisms of Action. Front. Pharmacol..

[76] Wang Y.-C., Wang X., Yu J., Ma F., Li Z., Zhou Y., Zeng S., Ma X., Li Y.-R., Neal A. (2021). Targeting monoamine oxidase A-regulated tumor-associated macrophage polarization for cancer immunotherapy. Nat. Commun..

[77] Breiman L., Friedman J.H., Olshen R.A., Stone C.J. (1984). Classification and Regression Trees.

[78] Baraldi P.G., Preti D., Tabrizi M.A., Fruttarolo F., Saponaro G., Baraldi S., Romagnoli R., Moorman A.R., Gessi S., Varani K. (2007). N(6)-[(hetero)aryl/(cyclo)alkyl-carbamoyl-methoxy-phenyl]-(2-chloro)-5′-N-ethylca rboxamido-adenosines: The first example of adenosine-related structures with potent agonist activity at the human A(2B) adenosine receptor. Bioorg. Med. Chem..

