# Supplementary material for: Pyridazinones and Structurally Related Derivatives with Anti-Inflammatory Activity
Source: Molecules. 2022 Jun 10;27(12):3749. doi: 10.3390/molecules27123749 (PMC9229294; doi:10.3390/molecules27123749)
Supplement: Supplementary file 1 [file molecules-27-03749-s001.zip › molecules-1761444-supplementary.pdf]

Supplementary Materials

for

Pyridazinones and Structurally Related Derivatives with Anti-inflammatory Activity

Niccolò Cantini[1,2,3#], Igor A. Schepetkin[1#], Nadezhda V. Danilenko[4], Andrei I. Khlebnikov[4], Letizia Crocetti[3], Maria Paola Giovannoni[3], Liliya N. Kirpotina[1], and Mark T. Quinn[1*]

[1] Department of Microbiology and Cell Biology, Montana State University, Bozeman, MT 59717, USA

[2]Department of Medicinal Chemistry, University of Antwerp, 2610 Antwerp, Belgium

[3] NEUROFARBA, Pharmaceutical and Nutraceutical Section, University of Florence, 50019 Sesto Fiorentino, Italy

[4] Kizhner Research Center, Tomsk Polytechnic University, Tomsk, Russia

Synthesis of Compounds 61 and 139

1. Chemistry

Synthesis of compound **61** is shown in **Scheme 1**. The starting material **179** [2] was alkylated with ethyl bromoacetate in the presence of potassium carbonate in dry acetonitrile at reflux, resulting in **180**. Subsequently, both the carboxy group and the amide at para position of the benzyl were hydrolyzed with sodium hydroxide 6N at 60°. The resulting acid **181** was finally reacted with 4-bromo-aniline to obtain the final product **61**, using ethyl chloroformate and triethylamine in dry tetrahydrofuran (THF).

Synthesis of compound **139** (AMC1) is shown in **Scheme 2**. The commercial product (**178**) was directly alkylated with N-(4-bromophenyl)-2-chloroacetamide, the synthesis of which is reported in the literature [1], with potassium carbonate in dry acetonitrile.

1.1. Scheme 1

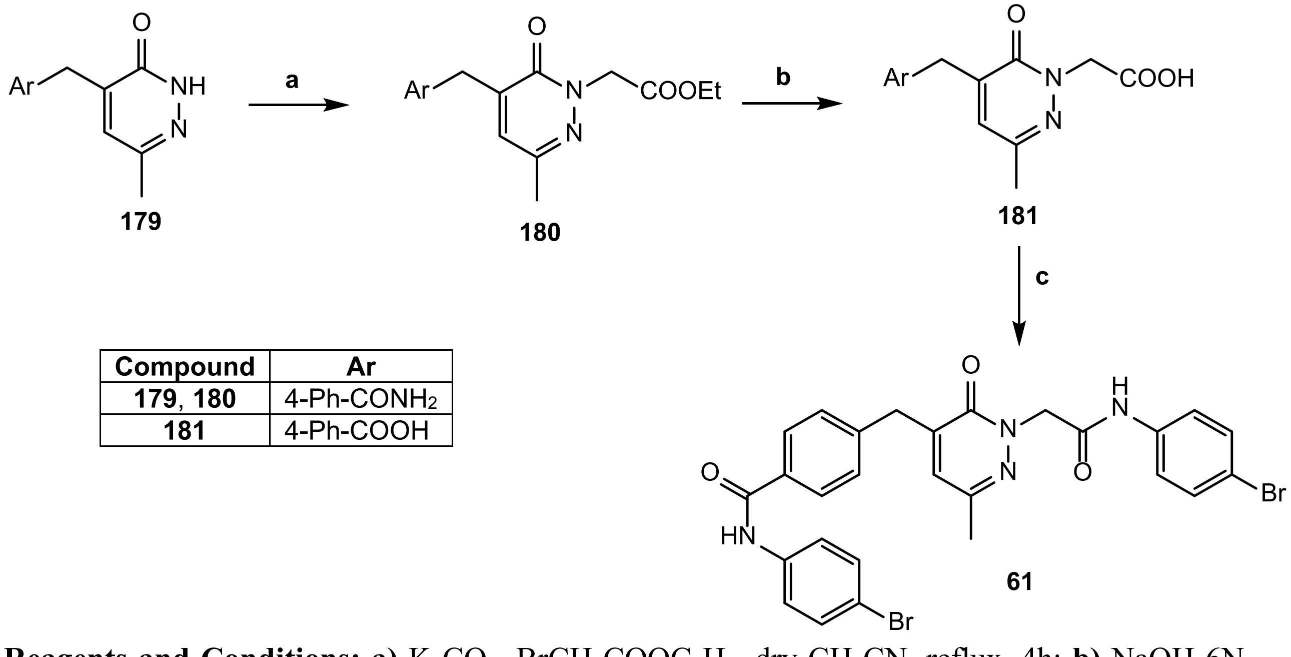

Compound	Ar
**179**, **180**	4-Ph-$CONH_2$
**181**	4-Ph-COOH

Reagents and Conditions: a) K_2CO_3, $BrCH_2COOC_2H_5$, dry CH_3CN, reflux, 4h; **b)** NaOH 6N, 60°C, 3h; **c)** 4-Bromoaniline, dry THF, $ClCOOC_2H_5$, Et_3N, 0°C, 3h.

1.2. Scheme 2

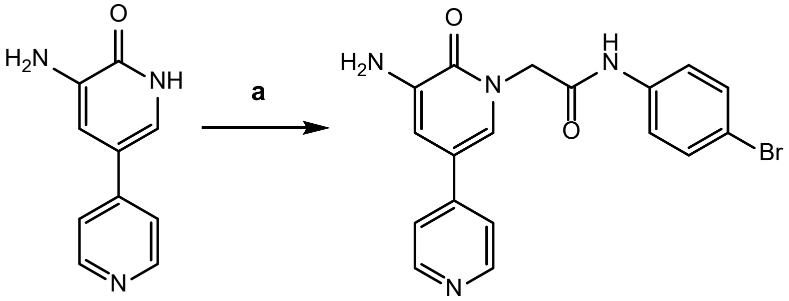

178 **139**

Reagents and Conditions: a) K_2CO_3, dry CH_3CN, N-(4-bromophenyl)-2-chloroacetamide, reflux, 3h.

2. Experimental section

All melting points were determined on a Büchi apparatus (New Castle, DE) and are uncorrected. Extracts were dried over Na_2SO_4, and the solvents were removed under reduced pressure. Merck F-254 commercial plates (Merck, Durham, NC) were used for analytical TLC to follow the course of reactions. Silica gel 60 (Merck 70-230 mesh, Merck, Durham, NC) was used for column chromatography. ^{1}H-NMR and ^{13}C-NMR spectra were recorded on an Avance 400 instrument (Bruker Biospin Version 002 with SGU, Bruker Inc., Billerica, MA). Chemical shifts (δ) are reported in ppm to the nearest 0.01 ppm using the solvent as an internal standard. Coupling constants (J values) are given in Hz and were calculated using TopSpin 4.0.8 software (Nicolet Instrument Corp., Madison, WI) and are rounded to the nearest 0.1 vHz. High resolution mass spectrometry (HRMS) analysis was performed with a Thermo Finnigan LTQ Orbitrap mass spectrometer equipped with an electrospray ionization source (ESI). The solvents used in HRMS were acetonitrile (Chromasolv grade) purchased from Sigma-Aldrich (Milan, Italy) and mQ water 18 MΩ cm, obtained from Millipore's Simplicity system (Milan, Italy). The accurate mass measure was performed by introducing sample solution (1.0 µg mL-1 in mQ water: acetonitrile 50:50) via syringe pump at 10 µL min-1, and the signal of the positive ions was acquired. The experimental conditions allowed monitoring of the protonated molecules ([M+H]+ species) with a proper dwell time to achieve 60.000 units of resolution at full width at half maximum (FWHM).

Microanalyses indicated by the symbols of the elements or functions were performed with a Perkin–Elmer 260 elemental analyzer (PerkinElmer, Inc., Waltham, MA) for C, H, and N, and the results were within ±0.4% of the theoretical values, unless otherwise stated. Reagents and starting material were commercially available.

N-(4-Bromophenyl)-4-((2-(2-((4-bromophenyl)amino)-2-oxoethyl)-6-methyl-3-oxo-2,3-dihydropyridazin-4-yl)methyl)benzamide (**61**). To a solution of 0.93 mmol of 4-[(2-(carboxymethyl)-6-methyl-3-oxo-2,3-dihydropyridazin-4-yl)methyl]benzoic acid (**181**) in 7 mL of anhydrous THF, 3.26 mmol of triethylamine was added, and the mixture was left at -7°C for 30'. Then, 1.02 mmol of ethyl chloroformate was added and the mixture was left for 1 h at 0°C. Finally, 1.86 mmol of 4-bromoaniline was added and the reaction was kept stirring at room temperature for 24h. After evaporation of the solvent, cold water was added (20 mL), and the pH was neutralized with 1N HCl. The suspension was extracted with CH_2Cl_2 (3 x 15 mL), dried over sodium sulfate and evaporated under vacuum. The crude compound 61 were purified by flash column chromatography using dichloromethane/methanol/ammonium hydroxide 95:5:0.5 as eluent. Yield = 5%; mp = 226-229°C. ^{1}H-NMR (400 MHz, DMSO-d_6) δ 2.31 (s, 3H, CH_3), 4.00 (s, 2H, CH_2-Ar), 4.93 (s, 2H, N-CH_2), 6.83 (s, 1H, CH), 7.40 (t, 6H, Ar), 7.51 (d, 2H, Ar), 7.57 (d, 2H, Ar), 7.84 (d, 2H, Ar), 8.83 (exch br s, 1H, NH). ^{13}C-NMR (100 MHz, DMSO-d6) δ 27.5 (CH_3), 42.5 (CH_2), 55.2 (CH_2), 121.9 (CH), 122.3 (C), 127.4 (CH), 129.1 (CH), 131.2 (C), 131.8 (CH), 136.9 (C), 137.5 (C), 139.7 (CH), 141.0 (C), 143.3 (C), 144.3 (C), 158.9 (C), 164.7 (C), 168.5 (C). ESI-MS calcd. for $C_{27}H_{22}Br_2N_4O_3$, 610,31, found m/z 609,01 [M+H]+. Anal. $C_{27}H_{22}Br_2N_4O_3$ (C, H, N).

2-(5-Amino-6-oxo-[3,4'-bipyridin]-1(6H)-yl)-N-(4-bromophenyl)acetamide (**139**). To a suspension of commercially available 5-amino-[3,4'-bipyridin]-6(1H)-one (**178**) (0.53 mmol) in 10 mL of acetonitrile, 0.53 mmol of N-(4-bromophenyl)-2-chloroacetamide and 1.06 mmol of K_2CO_3 were added, and the mixture was kept stirring at reflux for 3h. After cooling, the solvent

was evaporated and diluted with cold water. The precipitate was recovered by vacuum filtration, and the final compound 2 was purified by crystallization with ethanol. Yield = 88%; mp = 292-294°C. ^{1}H-NMR (400 MHz, DMSO-d$_6$) δ 4.79 (s, 2H, CH_2), 5.28 (exch br s, 2H, NH_2), 6.90 (s, 1H, Ar), 7.46 (m, 4H, Ar), 7.54 (m, 2H, Ar), 7.61 (s, 1H, Ar), 8.52 (m, 2H, Ar), 10.48 (exch br s, 1H, NH). ^{13}C-NMR (100 MHz, DMSO-d$_6$) δ 53.9 (CH_2), 109.4 (CH), 121.7 (CH), 122.3 (C), 123.3 (C), 131.8 (CH), 134.8 (CH), 137.5 (C), 143.3 (CH), 144.8 (C), 149.3 (C), 158.6 (C), 168.5 (C). ESI-MS calcd. for $C_{32}H_{27}BrN_4O_4$, 399.25, found m/z 399,04 [M+H]+. Anal. $C_{32}H_{27}BrN_4O_4$ (C, H, N).

Ethyl 2-(5-(4-carbamoylbenzyl)-3-methyl-6-oxopyridazin-1(6H)-yl)acetate (**180**). To a suspension of 4-[(6-methyl-3-oxo-2,3-dihydropyridazin-4-yl)methyl]benzamide (**179**) [2] (1.56 mmol) in 10 mL of acetonitrile, 2.34 mmol ethyl bromoacetate and 3.12 mmol of K_2CO_3 were added, and the mixture was kept stirring at reflux for 2h. After cooling, the solvent was evaporated and diluted with cold water. The precipitate was recovered by vacuum filtration, and the final compound 4 was purified by crystallization with ethanol. Yield = 78%; mp = 174-176°C. ^{1}H-NMR (400 MHz, $CDCl_3$) δ 1.31 (t, 3H, CH_2CH_3), 2.25 (s, 3H, 3-CH_3), 3.96 (s, 2H, CH_2-Ar), 4.26 (q, 2H, CH_2CH_3), 4.86 (s, 2H, N-CH_2), 6.70 (s, 1H, CH), 7.34 (s, 2H, Ar), 7.80 (d, 2H, Ar). ESI-MS calcd. for $C_{17}H_{19}N_3O_4$, 329,36, found m/z 330,14 [M+H]+. Anal. $C_{17}H_{19}N_3O_4$ (C, H, N).

4-((2-(Carboxymethyl)-6-methyl-3-oxo-2,3-dihydropyridazin-4-yl)methyl)benzoic acid (**181**). A suspension of 1.22 mmol of ethyl 2-[5-(4-carbamoylbenzyl)-3-methyl-6-oxopyridazin-1(6H)-yl]acetate (180) in 5 mL of 6N NaOH was heated to 60°C. After 2h, the mixture was cooled with ice, adjusted to an acidic pH with 6N HCl, and the precipitate formed was recovered by vacuum filtration. The final compound 5 was purified by crystallization with ethanol. Yield = 76%; mp = 225-227°C. ^{1}H-NMR (400 MHz, DMSO-d$_6$) δ 2.22 (s, 3H, CH_3), 3.87 (s, 2H, CH_2-Ar), 4.69 (s, 2H, N-CH_2), 7.15 (s, 1H, CH), 7.39 (d, 2H, Ar), 7.88 (d, 2H, Ar), 13.01

(exch br s, 1H, OH). ESI-MS calcd. for $C_{15}H_{14}N_2O_5$, 302,29, found m/z 303,09 [M+H]+. Anal. $C_{15}H_{14}N_2O_5$ (C, H, N).

References

1. Baraldi, P.G., et al., (2007) N(6)-[(hetero)aryl/(cyclo)alkyl-carbamoyl-methoxy-phenyl]-(2-chloro)-5'-N-ethylca rboxamido-adenosines: the first example of adenosine-related structures with potent agonist activity at the human A(2B) adenosine receptor. *Bioorg Med Chem* **15**: p. 2514-2527.
2. Giovannoni, M.P., et al. (2013) Further studies on 2-arylacetamide pyridazin-3(2H)-ones: design, synthesis and evaluation of 4,6-disubstituted analogs as formyl peptide receptors (FPRs) agonists. *Eur J Med Chem* **64**: 512-528.

Supplementary Table S1. Selected ADME parameters of the investigated compounds calculated with SwissADME web tool.

Compd.	MW	TPSA	iLOGP	XLOGP3	Silicos-IT Log P	Ali Solubility (mg/mL)
**1**	442.31	73.22	3.48	3.28	4.17	1.42E-02
**2**	442.31	73.22	3.55	3.28	4.17	1.42E-02
**3**	442.31	73.22	3.59	3.28	4.17	1.42E-02
**4**	397.85	73.22	3.39	3.22	4.14	1.48E-02
**5**	408.41	119.04	2.70	2.42	1.35	1.12E-02
**6**	423.46	91.68	3.62	2.53	3.64	3.35E-02
**7**	431.41	73.22	3.43	3.47	4.59	8.82E-03
**8**	504.38	73.22	3.58	4.94	5.24	3.07E-04
**9**	418.31	92.23	3.09	2.99	4.74	1.07E-02
**10**	484.34	90.29	3.43	3.94	4.35	1.41E-03
**11**	418.31	92.23	3.14	3.02	4.74	9.99E-03
**12**	442.26	90.29	3.06	2.59	3.20	3.24E-02
**13**	462.34	63.99	3.58	4.56	5.15	1.09E-03
**14**	419.52	73.22	3.78	4.26	4.9	1.30E-03
**15**	381.4	73.22	3.16	2.69	3.92	5.03E-02
**16**	363.41	73.22	3.14	2.59	3.49	6.08E-02
**17**	388.42	97.01	3.00	2.31	3.54	4.02E-02
**18**	510.42	73.22	3.85	5.36	5.34	1.14E-04
**19**	428.28	73.22	3.12	2.98	3.78	2.82E-02
**20**	503.33	73.22	3.53	3.71	4.86	5.80E-03
**21**	505.37	89.29	2.99	3.78	5.02	2.26E-03
**22**	519.33	82.45	3.51	3.21	4.54	1.26E-02
**23**	493.73	63.99	3.55	3.90	5.04	5.64E-03
**24**	409.5	98.52	3.31	3.10	4.12	5.96E-03
**25**	369.16	63.99	2.16	1.34	2.42	1.91E+00
**26**	337.17	90.01	2.15	0.91	1.42	1.39E+00
**27**	520.17	93.09	3.72	3.11	3.56	9.61E-03
**28**	329.37	63.99	2.52	2.87	3.06	4.41E-02
**29**	351.37	63.99	2.93	2.72	3.85	6.73E-02
**30**	412.28	63.99	3.22	3.31	4.11	1.93E-02
**31**	355.39	82.45	2.57	2.58	2.50	3.90E-02
**32**	377.39	82.45	3.07	2.43	3.30	5.92E-02
**33**	469.49	91.68	3.58	4.06	4.4	9.60E-04
**34**	443.47	73.22	3.54	4.35	4.98	1.11E-03
**35**	475.52	119.92	3.46	4.07	5.02	2.42E-04
**36**	449.5	101.46	3.56	4.36	5.6	2.80E-04
**37**	510.4	101.46	3.52	4.95	5.86	7.75E-05
**38**	473.5	82.45	3.56	4.32	5.05	8.12E-04
**39**	499.51	100.91	3.75	4.03	4.47	7.02E-04
**40**	534.4	82.45	4.40	4.91	5.31	2.24E-04
**41**	538.82	73.22	3.80	5.57	5.88	7.29E-05
**42**	477.91	73.22	3.84	4.98	5.62	2.65E-04
**43**	517.92	108.75	4.08	4.31	4.59	2.55E-04
**44**	532.39	90.29	3.91	4.92	5.31	1.49E-04
**45**	483.52	91.68	4.04	4.42	4.93	4.18E-04
**46**	457.5	73.22	3.90	4.71	5.51	4.83E-04
**47**	461.46	73.22	3.71	4.45	5.4	9.07E-04
**48**	487.48	91.68	3.86	4.16	4.82	7.85E-04
**49**	522.37	73.22	4.17	5.04	5.66	2.51E-04
**50**	549.42	85.69	4.24	4.77	4.09	2.75E-04
**51**	443.29	85.25	3.47	3.10	3.01	1.23E-02
**52**	471.3	102.32	3.59	2.39	2.95	3.11E-02

**53**	456.29	90.29	3.51	2.90	3.73	1.59E-02
**54**	518.4	73.22	3.58	4.87	5.64	3.73E-04
**55**	491.18	63.99	3.42	4.00	4.79	4.42E-03
**56**	472.33	82.45	3.68	3.25	4.25	1.04E-02
**57**	337.35	63.99	2.54	2.82	3.47	5.09E-02
**58**	458.37	89.29	2.98	3.82	4.74	1.87E-03
**59**	402.24	77.13	3.05	2.37	3.49	9.42E-02
**60**	413.27	76.88	2.78	2.24	3.55	1.34E-01
**61**	610.3	93.09	4.14	4.83	5.55	1.85E-04
**62**	455.3	107.08	2.78	2.18	3.34	3.95E-02
**63**	437.29	87.78	3.04	3.03	4.15	1.26E-02
**64**	430.27	63.99	3.22	3.41	4.53	1.59E-02
**65**	446.72	63.99	3.51	3.94	4.75	4.64E-03
**66**	480.28	63.99	3.45	4.19	5.2	2.74E-03
**67**	398.25	63.99	3.09	3.41	3.73	1.47E-02
**68**	336.18	63.99	2.72	1.75	2.63	6.54E-01
**69**	456.33	73.22	3.63	3.65	4.7	6.07E-03
**70**	398.25	63.99	2.92	3.01	3.73	3.82E-02
**71**	518.4	73.22	3.45	4.92	5.77	3.31E-04
**72**	434.29	110.32	2.95	1.72	1.65	9.66E-02
**73**	485.33	102.32	3.02	3.55	3.48	2.01E-03
**74**	403.23	118.69	1.67	0.87	1.98	4.56E-01
**75**	417.26	107.83	2.26	0.89	1.45	7.61E-01
**76**	523.38	103.07	3.25	3.08	3.01	6.41E-03
**77**	523.38	103.07	3.28	3.08	3.01	6.41E-03
**78**	470.36	73.22	3.95	4.09	5.1	2.18E-03
**79**	484.39	73.22	4.04	4.63	5.51	6.19E-04
**80**	498.41	73.22	3.97	5.17	5.91	1.75E-04
**81**	499.4	85.25	3.98	4.99	4.73	1.51E-04
**82**	443.29	85.25	3.24	3.1	3.01	1.23E-02
**83**	485.37	85.25	3.75	4.45	4.33	5.33E-04
**85**	456.33	64.43	4.08	3.46	4.13	1.46E-02
**89**	470.36	73.22	3.62	3.87	4.65	3.70E-03
**105**	398.88	53.35	4.21	4.63	5.31	1.33E-03
**106**	349.43	56.15	3.93	4.31	3.95	2.19E-03
**107**	398.88	53.35	4.16	4.63	5.31	1.33E-03
**108**	394.46	62.58	4.33	3.97	4.73	4.08E-03
**109**	408.45	79.65	3.78	3.92	4.28	2.09E-03
**110**	370.45	67.67	3.38	1.03	2.25	3.37E+00
**111**	456.33	73.22	3.97	3.18	4.57	1.86E-02
**112**	443.29	70.42	4.03	3.86	4.5	4.08E-03
**113**	471.35	85.25	3.94	3.08	3.8	1.37E-02
**114**	456.33	73.22	3.80	3.35	4.57	1.24E-02
**115**	443.29	85.25	3.81	2.71	3.01	3.11E-02
**116**	442.31	73.22	3.77	4.00	4.17	2.55E-03
**117**	428.28	73.22	3.93	2.98	3.78	2.82E-02
**118**	457.32	85.25	3.80	3.73	3.4	2.81E-03
**119**	458.37	88.24	3.72	3.88	5.63	1.70E-03
**120**	485.33	102.32	3.16	3.55	3.48	2.01E-03
**121**	371.17	61.77	2.46	1.00	2.82	4.83E+00
**133**	511.79	60.33	4.02	6.27	5.9	2.42E-05
**134**	449.34	43.26	3.34	5.03	5.19	9.40E-04
**135**	491.34	85.37	3.87	4.59	4.10	3.84E-04
**136**	476.32	73.34	3.75	4.87	4.88	3.41E-04
**137**	505.36	85.37	4.02	4.92	4.5	1.80E-04
**138**	458.37	89.41	3.48	4.58	5.17	3.02E-04

**139**	399.24	90.01	1.83	1.62	2.49	3.01E-01
**140**	482.33	93.35	3.18	3.41	4.47	4.30E-03
**141**	452.3	84.12	3.45	3.43	4.39	6.00E-03
**142**	418.24	101.19	2.90	2.22	3.11	4.38E-02
**143**	452.3	84.12	3.09	3.43	4.39	6.00E-03
**144**	542.38	115.73	3.39	3.31	3.97	2.08E-03
**145**	542.38	115.73	3.73	3.31	3.97	2.08E-03
**146**	547.4	102.32	3.28	3.48	4.54	2.67E-03
**147**	495.33	113.22	2.76	2.81	3.58	7.08E-03
**148**	495.33	113.22	2.58	2.81	3.58	7.08E-03
**149**	414.25	73.1	3.02	2.91	2.83	3.25E-02
**150**	430.25	82.33	2.88	2.52	2.37	5.48E-02
**151**	490.35	73.1	3.40	4.54	4.4	7.82E-04
**152**	366.21	73.1	2.77	1.88	1.99	3.36E-01
**153**	400.23	73.1	2.79	2.54	2.31	7.59E-02
**154**	472.33	82.33	3.61	3.49	3.68	5.92E-03
**155**	426.27	91.97	2.48	4.04	2.45	9.01E-04
**156**	426.27	91.97	2.63	4.04	2.45	9.01E-04
**157**	306.36	72.94	2.27	2.92	2.26	2.36E-02
**158**	491.38	68.18	3.30	5.8	4.52	4.90E-05
**159**	491.38	68.18	3.17	5.8	4.52	4.90E-05
**160**	425.28	79.94	3.13	3.68	3.75	3.80E-03
**161**	425.28	79.94	3.24	3.68	3.75	3.80E-03
**162**	401.26	68.18	2.37	3.77	2.41	5.11E-03
**163**	372.22	56.03	2.70	3.21	2.24	3.25E-02
**164**	493.35	77.29	3.80	4.93	3.09	2.53E-04
**165**	389.27	79.34	2.69	3.67	4.41	3.67E-03
**166**	419.29	88.57	3.07	3.64	4.45	2.72E-03
**167**	419.29	88.57	3.17	3.64	4.45	2.72E-03
**168**	423.71	79.34	3.24	4.3	5.04	8.86E-04
**169**	434.26	125.16	2.83	3.5	2.25	6.70E-04
**170**	423.71	79.34	3.21	4.30	5.04	8.86E-04
**171**	404.28	105.36	2.59	2.99	3.69	5.50E-03
**172**	369.23	96.41	2.58	2.31	3.74	3.93E-02
**173**	327.2	79.34	2.43	2.41	3.31	6.26E-02
**174**	386.24	63.99	2.67	2.86	3.69	5.30E-02
**175**	494.34	82.33	3.24	3.87	3.79	2.50E-03
**176**	358.19	63.99	2.5	2.48	2.67	1.22E-01
**177**	377.19	90.02	2.29	1.98	2.53	1.20E-01

Supplementary Table S2. Experimentally observed and predicted classes for inhibition of NF-κB activity by pyridazinones and related derivatives in according to classification tree analysis.

Compd.	Observed	Predicted	Terminal node	Compd.	Observed	Predicted	Terminal node
**1**	N.A.	N.A.	10	**75**	N.A.	N.A.	9
**2**	Active	N.A.	10	**76**	N.A.	N.A.	9
**3**	N.A.	N.A.	10	**77**	N.A.	N.A.	9
**4**	N.A.	N.A.	10	**78**	Active	Active	3
**5**	Active	Active	7	**79**	Active	Active	3
**6**	N.A.	N.A.	9	**80**	Active	Active	3
**7**	N.A.	N.A.	10	**81**	N.A.	Active	3
**8**	N.A.	Active	11	**82**	N.A.	N.A.	9
**9**	Active	N.A.	9	**83**	Active	Active	3
**10**	Active	Active	3	**85**	N.A.	N.A.	10
**11**	N.A.	N.A.	9	**89**	Active	Active	3
**12**	N.A.	N.A.	9	**105**	N.A.	N.A.	10
**13**	N.A.	Active	11	**106**	N.A.	N.A.	10
**14**	N.A.	N.A.	10	**107**	N.A.	N.A.	10
**15**	Active	N.A.	10	**108**	N.A.	N.A.	10
**16**	N.A.	N.A.	10	**109**	Active	N.A.	10
**17**	N.A.	N.A.	9	**110**	N.A.	N.A.	10
**18**	N.A.	Active	3	**111**	N.A.	N.A.	10
**19**	N.A.	N.A.	10	**112**	N.A.	N.A.	10
**20**	N.A.	N.A.	10	**113**	Active	Active	3
**21**	N.A.	N.A.	9	**114**	N.A.	N.A.	10
**22**	N.A.	N.A.	10	**115**	N.A.	N.A.	9
**23**	Active	N.A.	10	**116**	N.A.	N.A.	10
**24**	N.A.	N.A.	9	**117**	N.A.	N.A.	10
**25**	N.A.	N.A.	10	**118**	N.A.	N.A.	9
**26**	N.A.	N.A.	9	**119**	N.A.	N.A.	9
**27**	N.A.	N.A.	9	**120**	N.A.	N.A.	9
**28**	N.A.	N.A.	10	**121**	N.A.	N.A.	10
**29**	N.A.	N.A.	10	**133**	N.A.	N.A.	10
**30**	Active	N.A.	10	**134**	N.A.	N.A.	10
**31**	N.A.	N.A.	10	**135**	N.A.	N.A.	9
**32**	N.A.	N.A.	10	**136**	N.A.	N.A.	10
**33**	N.A.	N.A.	9	**137**	N.A.	N.A.	9
**34**	N.A.	Active	11	**138**	N.A.	N.A.	9
**35**	N.A.	N.A.	9	**139**	N.A.	N.A.	9
**36**	N.A.	N.A.	9	**140**	N.A.	N.A.	9
**37**	N.A.	N.A.	9	**141**	Active	Active	11
**38**	Active	Active	11	**142**	N.A.	N.A.	9
**39**	N.A.	N.A.	9	**143**	N.A.	Active	11
**40**	N.A.	Active	11	**144**	N.A.	N.A.	9
**41**	N.A.	Active	11	**145**	N.A.	N.A.	9
**42**	Active	Active	11	**146**	N.A.	N.A.	9
**43**	N.A.	N.A.	9	**147**	N.A.	N.A.	9
**44**	N.A.	N.A.	9	**148**	N.A.	N.A.	9
**45**	N.A.	N.A.	9	**149**	Active	Active	11
**46**	Active	Active	11	**150**	Active	Active	11
**47**	Active	Active	5	**151**	N.A.	Active	11
**48**	N.A.	Active	5	**152**	N.A.	N.A.	10
**49**	Active	Active	5	**153**	Active	Active	11
**50**	Active	Active	3	**154**	Active	Active	11
**51**	N.A.	N.A.	9	**155**	N.A.	N.A.	9
**52**	N.A.	N.A.	9	**156**	Active	N.A.	9

**53**	N.A.	N.A.	9	**157**	N.A.	N.A.	10
**54**	N.A.	Active	3	**158**	Active	N.A.	10
**55**	N.A.	N.A.	10	**159**	N.A.	N.A.	10
**56**	N.A.	N.A.	10	**160**	Active	N.A.	10
**57**	N.A.	Active	11	**161**	N.A.	N.A.	10
**58**	N.A.	N.A.	9	**162**	N.A.	N.A.	10
**59**	N.A.	N.A.	10	**163**	N.A.	N.A.	10
**60**	N.A.	N.A.	10	**164**	Active	Active	11
**61**	N.A.	N.A.	9	**165**	N.A.	N.A.	10
**62**	N.A.	N.A.	9	**166**	N.A.	N.A.	9
**63**	N.A.	N.A.	9	**167**	N.A.	N.A.	9
**64**	Active	Active	5	**168**	Active	N.A.	10
**65**	N.A.	N.A.	10	**169**	Active	Active	7
**66**	Active	Active	5	**170**	N.A.	N.A.	10
**67**	Active	Active	11	**171**	N.A.	N.A.	9
**68**	N.A.	N.A.	10	**172**	N.A.	N.A.	9
**69**	Active	N.A.	10	**173**	N.A.	N.A.	10
**70**	N.A.	N.A.	10	**174**	N.A.	N.A.	10
**71**	Active	Active	11	**175**	N.A.	Active	11
**72**	N.A.	N.A.	9	**176**	N.A.	N.A.	10
**73**	N.A.	N.A.	9	**177**	N.A.	N.A.	9
**74**	N.A.	N.A.	9				
